# Supplementary material for: COADVISE: covariate adjustment with variable selection in randomized controlled trials
Source: J R Stat Soc Ser A Stat Soc. 2025 Nov 4;189(3):1909–45. doi: 10.1093/jrsssa/qnaf171 (PMC13366223; doi:10.1093/jrsssa/qnaf171)
Supplement: qnaf171_Supplementary_Data [file qnaf171_supplementary_data.pdf]

Online Supplemental Material *for*

"COADVISE: Covariate Adjustment with Variable Selection in Randomized Controlled Trials"

Yi Liu, Ke Zhu, Larry Han, and Shu Yang

A Technical Proofs

In this section, we present our theoretical investigations into the efficiency gain achieved by the AIPW estimator for the ATE in RCT data. We adhere to the notation, setups, and assumptions established in the main text of the paper.

A.1 Minimizing the variance over a class of augmented estimators

First, consider a general class of augmented estimators for τ defined as follows:

$$\mathcal{G} = \{\widehat{\tau}^{\mathcal{G}}(g_0, g_1) : \mathbb{E}\{g_a(\mathbf{X})^2\} < \infty, a = 0, 1\}, \tag{A.1.1}$$

where

$$\widehat{\tau}^{\mathcal{G}} = \widehat{\tau}^{\mathcal{G}}(g_0, g_1) = \frac{1}{N}\sum_{i=1}^{N}\left\{\frac{A_iY_i}{\widehat{\pi}_1} - \frac{A_i - \widehat{\pi}_1}{\widehat{\pi}_1}g_1(\mathbf{X}_i)\right\} - \frac{1}{N}\sum_{i=1}^{N}\left\{\frac{(1-A_i)Y_i}{1-\widehat{\pi}_1} - \frac{\widehat{\pi}_1 - A_i}{1-\widehat{\pi}_1}g_0(\mathbf{X}_i)\right\} + o_p(N^{-1/2}),$$

with $\widehat{\pi}_1 = N_1/N$, the proportion of individuals with $A = 1$ in the sample.

We note that when $g_1(\mathbf{X}) = g_0(\mathbf{X}) = 0$, the estimator $\widehat{\tau}^{\mathcal{G}}$ reduces to $\widehat{\tau}_{\text{simple}}$. Furthermore, it is straightforward to show that for any choice of g_1 and g_0, $\widehat{\tau}^{\mathcal{G}} \to_p \tau$, using standard conditioning arguments and large sample theory.

The estimator $\widehat{\tau}^{\mathcal{G}}$ provides a general form of the AIPW estimator, as it consists of $\widehat{\tau}_{\text{simple}}$ augmented by functions g_0 and g_1. The term $o_p(N^{-1/2})$ indicates that two estimators in the class $\mathcal{G}$ differ by a negligible term that converges to 0 in probability with zero asymptotic variance. As can be observed, $\mathcal{G}$ is a large (uncountable) set, containing functions from an infinite-dimensional space.

When $g_a(\mathbf{X}) = \mathbb{E}(Y(a) \mid \mathbf{X})$, representing the fully specified model for $Y(a)$, $a = 0, 1$, the estimator $\widehat{\tau}^{\mathcal{G}}$ achieves the semiparametric efficiency bound (Hahn, 1998; Hirano et al., 2003). However, since the true model of $Y(a)$ as a function of $\mathbf{X}$ is unknown, achieving this bound is generally not expected. Nevertheless, we can still improve efficiency through "appropriate augmentation," even without precise model specification,

compared to $\widehat{\tau}_{\text{simple}}$. This is our objective in leveraging covariate information to gain efficiency (Bannick et al., 2023).

In practice, we focus on a subclass of $\mathcal{G}$, denoted as $\mathcal{G}^g_{\boldsymbol{\beta}} \subset \mathcal{G}$, where $g_a(\mathbf{X}) = g(\mathbf{X}'\boldsymbol{\beta}_a)$ follows a generalized linear model (GLM) for $Y(a)$, for $a = 0, 1$. This subclass is characterized by the given function g, for all vectors $\boldsymbol{\beta} \in \mathcal{R}^p$ such that $\mathbb{E}\{g(\mathbf{X}'\boldsymbol{\beta})^2\} < \infty$. If the true model for $Y(a)$ belongs to $\mathcal{G}^g_{\boldsymbol{\beta}}$, then there exists a fixed $\boldsymbol{\beta}^0_a \in \mathcal{R}^p$ such that $g(\mathbf{X}'\boldsymbol{\beta}^0_a) = \mathbb{E}(Y(a) \mid \mathbf{X})$. This subclass $\mathcal{G}^g_{\boldsymbol{\beta}}$ has practical appeal because, in many applications, we often fit both $Y(0)$ and $Y(1)$ using the same GLM with an appropriate link function g.

Next, we denote an AIPW estimator belonging to $\mathcal{G}^g_{\boldsymbol{\beta}}$ by $\widehat{\tau}^g$, highlighting the dependence on the link function g. Using argument $\mathbb{V}(\cdot) = \mathbb{E}\{\mathbb{V}(\cdot \mid \mathbf{X}, Y(0), Y(1))\} + \mathbb{V}\{\mathbb{E}(\cdot \mid \mathbf{X}, Y(0), Y(1))\}$, we have the asymptotic variance of $\widehat{\tau}^g$ as follows:

$$
\begin{aligned}
\mathbb{V}(\widehat{\tau}^g) &= \mathbb{V}\left\{\frac{AY}{\pi_1} - \frac{A-\pi_1}{\pi_1} g(\mathbf{X}'\boldsymbol{\beta}^*_1) - \frac{(1-A)Y}{1-\pi_1} + \frac{\pi_1 - A}{1-\pi_1} g(\mathbf{X}'\boldsymbol{\beta}^*_0)\right\} \\
&= \mathbb{V}\left\{\frac{AY(1)}{\pi_1} - \frac{A-\pi_1}{\pi_1} g(\mathbf{X}'\boldsymbol{\beta}^*_1) - \frac{(1-A)Y(0)}{1-\pi_1} + \frac{\pi_1 - A}{1-\pi_1} g(\mathbf{X}'\boldsymbol{\beta}^*_0)\right\} \\
&= \mathbb{E}\left\{\frac{1-\pi_1}{\pi_1}\{Y(1) - g(\mathbf{X}'\boldsymbol{\beta}^*_1)\}^2 + \frac{\pi_1}{1-\pi_1}\{Y(0) - g(\mathbf{X}'\boldsymbol{\beta}^*_0)\}^2\right\} + \mathbb{V}\{Y(1) - Y(0)\}, \qquad \text{(A.1.2)}
\end{aligned}
$$

where $\boldsymbol{\beta}^*_a$ is the probability limit of $\widehat{\boldsymbol{\beta}}_a$, and $\widehat{\boldsymbol{\beta}}_a$ is an estimator (e.g., the ordinary least square [OLS] estimator) used to estimate $\boldsymbol{\beta}_a$ $(a = 0, 1)$.

Therefore, to minimize (A.1.2) over choices of $(\boldsymbol{\beta}^{*\prime}_0, \boldsymbol{\beta}^{*\prime}_1)'$, it is equivalent to minimize

$$
\mathbb{E}\left\{\frac{1-\pi_1}{\pi_1}\{Y(1) - g(\mathbf{X}'\boldsymbol{\beta}^*_1)\}^2 + \frac{\pi_1}{1-\pi_1}\{Y(0) - g(\mathbf{X}'\boldsymbol{\beta}^*_0)\}^2\right\}. \qquad \text{(A.1.3)}
$$

Then, it is natural to consider setting the derivative with respect to $(\boldsymbol{\beta}^{*\prime}_0, \boldsymbol{\beta}^{*\prime}_1)'$ to 0, i.e., to solve equations

$$
\begin{aligned}
0 &= \frac{1-\pi_1}{\pi_1}\mathbb{E}\left\{g_{\boldsymbol{\beta}_1}(\mathbf{X}'\boldsymbol{\beta}^*_1)[Y(1) - g(\mathbf{X}'\boldsymbol{\beta}^*_1)]\right\}, \\
0 &= \frac{\pi_1}{1-\pi_1}\mathbb{E}\left\{g_{\boldsymbol{\beta}_0}(\mathbf{X}'\boldsymbol{\beta}^*_0)[Y(0) - g(\mathbf{X}'\boldsymbol{\beta}^*_0)]\right\}, \qquad \text{(A.1.4)}
\end{aligned}
$$

where $g_{\boldsymbol{\beta}}(\mathbf{X}'\boldsymbol{\beta}^*)$ denotes $\left.\frac{\partial}{\partial \boldsymbol{\beta}} g(\mathbf{X}'\boldsymbol{\beta})\right|_{\boldsymbol{\beta}=\boldsymbol{\beta}^*}$.

Observe that the solution of the following equation, say, $\widehat{\boldsymbol{\beta}}_a$,

$$
\frac{1}{N}\sum_{i=1}^{N} I(A_i = a) g_{\boldsymbol{\beta}_a}(\mathbf{X}'\boldsymbol{\beta}_a)[Y_i - g(\mathbf{X}'_i\boldsymbol{\beta}_a)] = 0, \qquad \text{(A.1.5)}
$$

converges to the solution of (A.1.4), for $a = 0, 1$. This is because when the outcome model is possibly

misspecified, i.e, when $g(\mathbf{X}'\boldsymbol{\beta}_a) \neq \mathbb{E}(Y(a) \mid \mathbf{X})$, the left-hand side of the above equation converges in probability to

$$\pi_a \mathbb{E}\left\{g_{\boldsymbol{\beta}_a}(\mathbf{X}'\boldsymbol{\beta}_a)[\mathbb{E}(Y(a) \mid \mathbf{X}) - g(\mathbf{X}'\boldsymbol{\beta}_a)]\right\} = \pi_a \mathbb{E}\left\{g_{\boldsymbol{\beta}_a}(\mathbf{X}'\boldsymbol{\beta}_a)[Y(a) - g(\mathbf{X}'\boldsymbol{\beta}_a)]\right\}, \tag{A.1.6}$$

for $a = 0, 1$, which differs from (A.1.4) only by a constant. Therefore, setting (A.1.6) to be 0 results in the same solution to (A.1.4). We refer to this $\widehat{\boldsymbol{\beta}}_a$ as the OLS estimator in the following text.

Moreover, to verify whether solving (A.1.4) always uniquely minimizes (A.1.3), we check if the second-order derivative of (A.1.3) with respect to $(\boldsymbol{\beta}_0^{*\prime}, \boldsymbol{\beta}_1^{*\prime})'$ is semi-positive definite (a sufficient but not necessary condition). The second-order derivative can be expressed as $\text{diag}(\boldsymbol{\Sigma}_0, \boldsymbol{\Sigma}_1)$ (diagonal matrix of two blocks), where

$$\begin{aligned}\boldsymbol{\Sigma}_a &= 2\frac{1-\pi_a}{\pi_a}\mathbb{E}\left\{-\{Y(a) - g(\mathbf{X}'\boldsymbol{\beta}_a^*)\}g_{\boldsymbol{\beta}_a\boldsymbol{\beta}_a}(\mathbf{X}'\boldsymbol{\beta}_a^*) + g_{\boldsymbol{\beta}_a}(\mathbf{X}'\boldsymbol{\beta}_a^*)g_{\boldsymbol{\beta}_a}(\mathbf{X}'\boldsymbol{\beta}_a^*)'\right\} \\ &= 2\frac{1-\pi_a}{\pi_a}\mathbb{E}\left\{g_{\boldsymbol{\beta}_a}(\mathbf{X}'\boldsymbol{\beta}_a^*)g_{\boldsymbol{\beta}_a}(\mathbf{X}'\boldsymbol{\beta}_a^*)'\right\} - 2\frac{1-\pi_a}{\pi_a}\mathbb{E}\left\{\{\mathbb{E}(Y(a) \mid \mathbf{X}) - g(\mathbf{X}'\boldsymbol{\beta}_a^*)\}g_{\boldsymbol{\beta}_a\boldsymbol{\beta}_a}(\mathbf{X}'\boldsymbol{\beta}_a^*)\right\},\end{aligned} \tag{A.1.7}$$

for $a = 0, 1$, where $g_{\boldsymbol{\beta}\boldsymbol{\beta}}(\mathbf{X}'\boldsymbol{\beta}^*)$ denotes $\left.\frac{\partial^2}{\partial\boldsymbol{\beta}\partial\boldsymbol{\beta}'}g(\mathbf{X}'\boldsymbol{\beta})\right|_{\boldsymbol{\beta}=\boldsymbol{\beta}^*}$. We note that the first term of (A.1.7) is always positive definite, while for the second term, further discussion is provided in Sections A.2 and A.3.

A.2 Guaranteed efficiency gain using linear models

Consider the special case where $g(\mathbf{X}'\boldsymbol{\beta}_a) = \mathbf{X}'\boldsymbol{\beta}_a$, the identical link function. In this case, the second term in (A.1.7) is always zero because $g_{\boldsymbol{\beta}_a\boldsymbol{\beta}_a}(\mathbf{X}'\boldsymbol{\beta}_a^*) = \mathbf{0}$. Thus, the minimum of (A.1.3) is always achieved by the OLS estimator $\widehat{\boldsymbol{\beta}}_a$ for $\boldsymbol{\beta}_a$. The efficiency gain is guaranteed, as $\widehat{\tau}_{\text{simple}} \in \mathcal{G}_{\boldsymbol{\beta}}^g$ by setting $\boldsymbol{\beta}_0 = \boldsymbol{\beta}_1 = \mathbf{0}$. Consequently, the estimator in the class $\mathcal{G}_{\boldsymbol{\beta}}^g$, which minimizes the variance, must be at least as efficient as $\widehat{\tau}_{\text{simple}}$.

Furthermore, this result can easily be extended situations where a variable selection step is performed before AIPW estimation. Suppose we select s variables from $\mathbf{X}$ for $Y(a)$ model, where $0 < s < p$. When fitting the OLS estimator, the only difference is that $\boldsymbol{\beta} \in \mathcal{R}^s$ instead of $\mathcal{R}^p$ for estimators in $\mathcal{G}_g^{\boldsymbol{\beta}}$. Here, $\widehat{\tau}_{\text{simple}}$ remains a special case of estimators in $\mathcal{G}_g^{\boldsymbol{\beta}}$. Therefore, the proof remains straightforward.

Moreover, this illustrates that with more covariates (i.e., higher dimensions of $\boldsymbol{\beta}_a$), there is a greater opportunity to achieve higher efficiency. Without loss of generality, assume the first s variables of $\mathbf{X}$ are selected. We can view the lower dimension s as $\boldsymbol{\beta}_a \in \mathcal{R}^p$ where $\beta_a^{(s+1)} = \cdots = \beta_a^{(p)} = 0$ are fixed, with $\beta_a^{(j)}$ denoting the j-th element of $\boldsymbol{\beta}_a$. Therefore, the class of estimators $\mathcal{G}_g^{\boldsymbol{\beta}}$ when $\boldsymbol{\beta}_a \in \mathcal{R}^s$ can be viewed as a subset of $\mathcal{G}_g^{\boldsymbol{\beta}}$ when $\boldsymbol{\beta}_a \in \mathcal{R}^p$. Naturally, the variance by a minimizer found from a larger class must

be at least as low as that by a minimizer found from a smaller subclass. However, when the sample size is not sufficiently large, including many covariates can negatively impact the finite-sample performance of the estimator, as all of the aforementioned results are based on large-sample asymptotics.

A.3 Conditions on efficiency gain under a nonlinear GLM

nvestigating efficiency gains with a general link function g is more challenging due to several factors: (i) the usual maximum likelihood estimator (MLE) for regression coefficients in a general GLM is not obtained by solving (A.1.5) to minimize the squared loss in (A.1.3); (ii) for a general link function g, the second-order derivative (A.1.7) is not always positive-definite; (iii) $\widehat{\tau}_{\text{simple}}$ may not even belong to $\mathcal{G}^g_{\boldsymbol{\beta}}$ for certain link functions.

For (iii), consider logistic regression. The logistic link function is given by $g(u) = \{1+e^{-u}\}^{-1} > 0$, which implies that the class $\mathcal{G}^g_{\boldsymbol{\beta}}$, defined by the logistic function g, does not include $\widehat{\tau}_{\text{simple}}$. This is because the influence function of $\widehat{\tau}_{\text{simple}}$ cannot be approximated by any influence functions of estimators in $\mathcal{G}^g_{\boldsymbol{\beta}}$ (in the sense that the difference between the two influence functions can be $O_p(N^{-r})$ with an $r \geq 1/2$). Achieving such an approximation would require $g(\mathbf{X}'\boldsymbol{\beta}^*_a) \to_p 0$ for all $\mathbf{X} \in \mathcal{X}$, which in trun would imply that there exists some fixed $\boldsymbol{\beta}^*_a$ such that $\mathbf{X}'\boldsymbol{\beta}^*_a \to_p -\infty$ for all $\mathbf{X} \in \mathcal{X}$. Consequently, $\widehat{\tau}_{\text{simple}}$ cannot even lie on the boundary (or closure) of $\mathcal{G}^g_{\boldsymbol{\beta}}$.

To further explore the conditions for efficiency gain, we examine the difference between the asymptotic variances of $\widehat{\tau}_{\text{simple}}$ and $\widehat{\tau}^g$, given by:

$$\begin{aligned}
\mathbb{V}(\widehat{\tau}_{\text{simple}}) - \mathbb{V}(\widehat{\tau}^g) &= \mathbb{E}\left\{\frac{1-\pi_1}{\pi_1}Y(1)^2 + \frac{\pi_1}{1-\pi_1}Y(0)^2\right\} - \mathbb{E}\left\{\frac{1-\pi_1}{\pi_1}\{Y(1)-g(\mathbf{X}'\boldsymbol{\beta}^*_1)\}^2 + \frac{\pi_1}{1-\pi_1}\{Y(0)-g(\mathbf{X}'\boldsymbol{\beta}^*_0)\}^2\right\} \\
&= \mathbb{E}\left\{\frac{1-\pi_1}{\pi_1}g(\mathbf{X}'\boldsymbol{\beta}_1)\{2Y(1)-g(\mathbf{X}'\boldsymbol{\beta}_1)\} + \frac{\pi_1}{1-\pi_1}g(\mathbf{X}'\boldsymbol{\beta}_0)\{2Y(0)-g(\mathbf{X}'\boldsymbol{\beta}_0)\}\right\} \\
&= \frac{1-\pi_1}{\pi_1}\underbrace{\mathbb{E}\left\{g(\mathbf{X}'\boldsymbol{\beta}_1)[2Y(1)-g(\mathbf{X}'\boldsymbol{\beta}_1)]\right\}}_{I_1} + \frac{\pi_1}{1-\pi_1}\underbrace{\mathbb{E}\left\{g(\mathbf{X}'\boldsymbol{\beta}_0)[2Y(0)-g(\mathbf{X}'\boldsymbol{\beta}_0)]\right\}}_{I_0}.
\end{aligned} \tag{A.3.1}$$

For both $a = 0, 1$, we note that $I_a = \mathbb{E}\left\{g(\mathbf{X}'\boldsymbol{\beta}_a)[2Y(a)-g(\mathbf{X}'\boldsymbol{\beta}_a)]\right\} = \mathbb{E}\left\{g(\mathbf{X}'\boldsymbol{\beta}_a)[2\mathbb{E}(Y(a) \mid \mathbf{X})-g(\mathbf{X}'\boldsymbol{\beta}_a)]\right\} = \mathbb{E}\{I_a(\mathbf{X})\}$, where $I_a(\mathbf{X}) = g(\mathbf{X}'\boldsymbol{\beta}_a)[2\mathbb{E}(Y(a) \mid \mathbf{X}) - g(\mathbf{X}'\boldsymbol{\beta}_a)]$. Therefore, $I_a(\mathbf{X})$ achieves its maximum when $g(\mathbf{X}'\boldsymbol{\beta}_a) = \mathbb{E}(Y(a) \mid \mathbf{X})$. This implies that when $g(\mathbf{X}'\boldsymbol{\beta}_a)$ is the fully specified model for $\mathbb{E}(Y(a) \mid \mathbf{X})$, $\mathbb{V}(\widehat{\tau}_{\text{simple}}) - \mathbb{V}(\widehat{\tau}^g)$ reaches its maximal value $\mathbb{E}\{\mathbb{E}\{Y(a)^2 \mid \mathbf{X}\}\} = \mathbb{E}\{Y(a)^2\}$, corresponding to the highest efficiency gain. Additionally, in this case, the second term in the (A.1.7) of the squared loss is always zero, which ensures $\boldsymbol{\beta}^*_a$ is the variance minimizer. However, this is typically unrealistic in practice, and if the model is not well-specified, it is possible that (A.3.1) can be negative. In the following, we outline some

conditions for achieving efficiency gains.

Condition A.3.1. *For a nonlinear link function g, with probability 1, and there exists some $\boldsymbol{\beta}_a^*$ $(a = 0, 1)$, we have: (a) the sign of $g(\mathbf{X}'\boldsymbol{\beta}_a^*)$ must match that of $\mathbb{E}(Y(a) \mid \mathbf{X})$; and (b) $|g(\mathbf{X}'\boldsymbol{\beta}_a^*)| \leq 2|\mathbb{E}(Y(a) \mid \mathbf{X})|$.*

If Condition A.3.1 holds, then it is clear that (A.3.1) is always non-negative. This condition may be mild for some positive categorical outcomes. In the following illustration, we consider logistic regression as a more specific example, as it is commonly used in binary outcome modelling. For both $a = 0, 1$, we further write

$$\begin{aligned} g_{\boldsymbol{\beta}_a}(\mathbf{X}'\boldsymbol{\beta}_a^*) &= \frac{\exp(-\mathbf{X}'\boldsymbol{\beta}_a^*)}{\{1+\exp(-\mathbf{X}'\boldsymbol{\beta}_a^*)\}^2}\mathbf{X}' = g(\mathbf{X}'\boldsymbol{\beta}_a^*)\{1-g(\mathbf{X}'\boldsymbol{\beta}_a^*)\}\mathbf{X}', \\ g_{\boldsymbol{\beta}_a\boldsymbol{\beta}_a}(\mathbf{X}'\boldsymbol{\beta}_a^*) &= \frac{\exp(-\mathbf{X}'\boldsymbol{\beta}_a^*)\{\exp(-\mathbf{X}'\boldsymbol{\beta}_a^*)-1\}}{\{1+\exp(-\mathbf{X}'\boldsymbol{\beta}_a^*)\}^3}\mathbf{X}\mathbf{X}' = g(\mathbf{X}'\boldsymbol{\beta}_a^*)\{1-g(\mathbf{X}'\boldsymbol{\beta}_a^*)\}\{1-2g(\mathbf{X}'\boldsymbol{\beta}_a^*)\}\mathbf{X}\mathbf{X}'. \end{aligned} \tag{A.3.2}$$

Therefore, for any general $\mathbf{X} \neq \mathbf{0}$, we have $g_{\boldsymbol{\beta}_a}(\mathbf{X}'\boldsymbol{\beta}_a^*) \neq \mathbf{0}$, since the logistic function satisfies $0 < g(\mathbf{X}'\boldsymbol{\beta}_a^*) < 1$. Additionally, $g_{\boldsymbol{\beta}_a\boldsymbol{\beta}_a}(\mathbf{X}'\boldsymbol{\beta}_a^*) = \mathbf{0}$ only when $\boldsymbol{\beta}_a^* = \mathbf{0}$ (so $g(\mathbf{X}'\boldsymbol{\beta}_a^*) = 1/2$); for other $\boldsymbol{\beta}_a^*$, the sign of $g_{\boldsymbol{\beta}_a\boldsymbol{\beta}_a}(\mathbf{X}'\boldsymbol{\beta}_a^*)$ can be either positive or negative. We now introduce Condition A.3.2 for using logistic regression to achieve efficiency gain.

Condition A.3.2 (Logistic outcome models)**.** *(i) There exists a constant $\delta > 0$ such that $\mathbb{E}(Y(1) \mid \mathbf{X}) = 1 - \mathbb{E}(Y(0) \mid \mathbf{X}) \geq \delta$ with probability 1 over $\mathbf{X} \in \mathcal{X}$; (ii) For logistic regression model $g(\mathbf{X}'\boldsymbol{\beta}_a^*)$ we assigned to $Y(a)$, $|f_a(\mathbf{X}) - g(\mathbf{X}'\boldsymbol{\beta}_a^*)| < g(\mathbf{X}'\boldsymbol{\beta}_a^*)\{1-g(\mathbf{X}'\boldsymbol{\beta}_a^*)\}|1-2g(\mathbf{X}'\boldsymbol{\beta}_a^*)|^{-1}$ with probability 1.*

If Condition A.3.2 is satisfied, it is straightforward to verify that the OLS estimator $\widehat{\boldsymbol{\beta}}_a$ minimizes the variance. We also have additional intuitions about Condition A.3.2 as follows. Condition A.3.2(i) requires the true proportion of positive responses under both treatment and control is non-zero, which is a mild assumption for many binary outcomes in practice. In Condition A.3.2(ii), the left-hand side of the inequality in (ii) can be interpreted as the (absolute local) bias of the model $g(\mathbf{X}'\boldsymbol{\beta}_a)$ at $\mathbf{X}$. This means that if the bias can be controlled by the right-hand side almost surely, along with (i), the proof in Section A.2 regarding the minimizer still applies to logistic regression.

Figure A.3.1 illustrates the right-hand side of inequality in (ii). From the figure, if $\boldsymbol{\beta}_a^*$ is such that $g(\mathbf{X}'\boldsymbol{\beta}^*) \approx 1/2$, the (absolute value of) bias is allowed to be relatively large. However, when $g(\mathbf{X}'\boldsymbol{\beta}^*)$ is either smaller or larger, the bias must be smaller, approaching zero when $g(\mathbf{X}'\boldsymbol{\beta}^*) \approx 0$ or 1. In other words, when the true proportion of $Y(a)$ is very small or vary large, the model must better approximate the true value. But when the true proportion is around 50%, more error in the posited model is acceptable. The intuition is when the model $g(\mathbf{X}'\boldsymbol{\beta}^*)$ approaches a medium value, the uncertainty regarding whether the true $Y(a)$ is 0 or 1 increases.

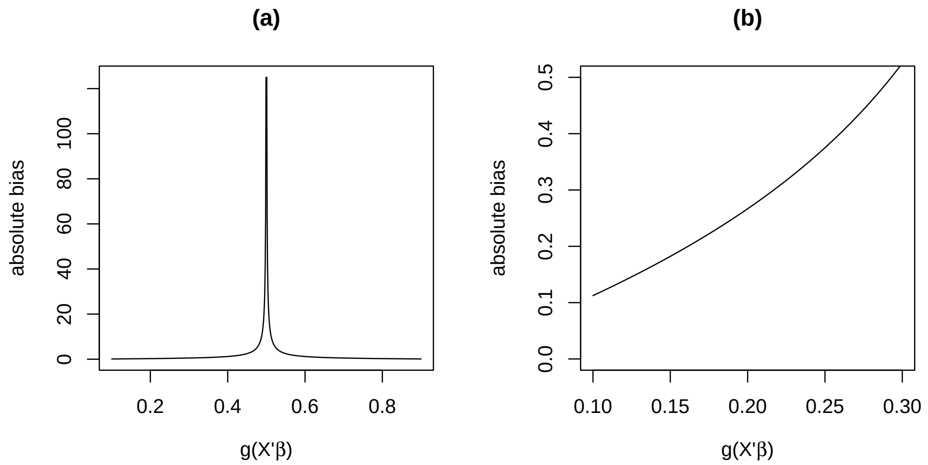

Figure A.3.1: An illustration for the bound of absolute bias in Condition A.3.2(ii) (right-hand side of the inequality). Panel (a) provides the absolute biases over $g(\mathbf{X}'\boldsymbol{\beta}) \in [0.1, 0.9]$, which ranges from $[0.11, \infty)$. When $g(\mathbf{X}'\boldsymbol{\beta}) = 0.5$, the absolute bias is actually ∞; Panel (b) provides a further look for $g(\mathbf{X}'\boldsymbol{\beta}) \in [0.1, 0.3]$.

Moreover, it may be natural to consider an alternative coefficient estimation strategy to gain efficiency without requiring the conditions mentioned above for a general GLM. Revisiting (A.3.1), for $a = 0, 1$, write

$$I_a = \mathbb{E}\{g(\mathbf{X}'\boldsymbol{\beta}_a)[2Y(a) - 2g(\mathbf{X}'\boldsymbol{\beta}_a) + g(\mathbf{X}'\boldsymbol{\beta}_a)]\} = 2\mathbb{E}\{g(\mathbf{X}'\boldsymbol{\beta}_a)[Y(a) - g(\mathbf{X}'\boldsymbol{\beta}_a)]\} + \mathbb{E}\{g(\mathbf{X}'\boldsymbol{\beta}_a)^2\}.$$

Therefore, to ensure $I_a \geq 0$, consider $\widetilde{\boldsymbol{\beta}}_a$ as the solution to the following joint equations

$$\frac{1}{N}\sum_{i=1}^{N} I(A_i = a) g(\mathbf{X}_i'\boldsymbol{\beta}_a)[Y_i - g(\mathbf{X}_i'\boldsymbol{\beta}_a)]\mathbf{X}' = \mathbf{0}. \tag{A.3.3}$$

Here $\mathbf{X}$ should include an intercept term so that setting (A.3.3) to zero implies $\frac{1}{N}\sum_{i=1}^{N} I(A_i = a) g(\mathbf{X}_i'\boldsymbol{\beta}_a)[Y_i - g(\mathbf{X}_i'\boldsymbol{\beta}_a)] = 0$. Therefore, the probability limit of $\widetilde{\boldsymbol{\beta}}_a$, denoted by $\boldsymbol{\beta}_a^{**}$, can satisfy $\mathbb{E}\{g(\mathbf{X}'\boldsymbol{\beta}_a^{**})[Y(a) - g(\mathbf{X}'\boldsymbol{\beta}_a^{**})]\} = 0$, and thus, for $a = 0, 1$, we have

$$I_a = 2\mathbb{E}\{g(\mathbf{X}'\boldsymbol{\beta}_a^{**})[Y(a) - g(\mathbf{X}'\boldsymbol{\beta}_a^{**})]\} + \mathbb{E}\{g(\mathbf{X}'\boldsymbol{\beta}_a^{**})^2\} = \mathbb{E}\{g(\mathbf{X}'\boldsymbol{\beta}_a^{**})^2\} \geq 0.$$

However, if we plug this $\boldsymbol{\beta}_a^{**}$ into (A.1.4), in general, $\mathbb{E}\{g_{\boldsymbol{\beta}_a}(\mathbf{X}'\boldsymbol{\beta}_a^{**})[Y(a) - g(\mathbf{X}'\boldsymbol{\beta}_a^{**})]\} \neq 0$, meaning that $\widetilde{\boldsymbol{\beta}}_a$ is not the global (optimal) variance minimizer among all $\widehat{\tau}^g \in \mathcal{G}_{\boldsymbol{\beta}}^g$. Another issue with this approach is that $\widetilde{\boldsymbol{\beta}}_a$ is not the MLE for the regression coefficients of a nonlinear GLM, so it does not guarantee unbiased predictions of the outcomes (Van Lancker et al., 2024). Finally, in practice, solving (A.3.3) can be unstable. For example, in logistic regression, a $\widetilde{\boldsymbol{\beta}}_a$ with some large elements can make $g(\mathbf{X}'\widetilde{\boldsymbol{\beta}}_a) \approx 0$, but this $\widetilde{\boldsymbol{\beta}}_a$ might

deviate significantly from the true value, leading to poor statistical properties. For these reasons, we did not incorporate this approach into our framework.

B Additional Simulation Details

B.1 Additional details of the data generating process

Recall that we generate the binary treatment assignment by $A \sim \text{Bern}(0.5)$, and we consider two sets of covariates $\mathbf{X} = (X_1, \ldots, X_5)'$ and $\mathbf{V} = (V_1, \ldots, V_{50})'$, where $(X_1, X_2) \sim \mathcal{N}_2(\boldsymbol{\mu}_X, \boldsymbol{\Sigma}_X)$ is a bivariate normal distribution with $\boldsymbol{\mu}_X = (0, 0)'$, $\boldsymbol{\Sigma}_X = \begin{pmatrix} 1 & 0.8 \\ 0.8 & 1 \end{pmatrix}$, $X_3 \sim \mathcal{N}(0, 1)$, $X_4 \sim t_{df=10}$, and $X_5 \sim \text{Bin}(10, 0.2) - 2$. Additionally, $\mathbf{V} \sim \mathcal{N}_{50}(\boldsymbol{\mu}_V, \boldsymbol{\Sigma}_V)$, where $\boldsymbol{\mu}_V = (\underbrace{1, \ldots, 1}_{\text{50 elements}})'$, and $\boldsymbol{\Sigma}_V$ is the correlation matrix of matrix $\boldsymbol{B}$, with

$$\boldsymbol{B} = \underbrace{\begin{pmatrix} 0.10 & 0.10 & \ldots & 0.10 \\ 0.11 & 0.11 & \ldots & 0.11 \\ \vdots & \vdots & \ddots & \vdots \\ 0.59 & 0.59 & \ldots & 0.59 \end{pmatrix}}_{\text{50 columns}} + 2\boldsymbol{I}_{50},$$

where $\boldsymbol{I}_{50}$ is the 50×50 identity matrix.

We specify the following two models for potential outcomes:

$$\text{Continuous outcome: } Y(a) = 30 + 20\mathbf{X}'\boldsymbol{\beta}_0 + a\delta(\mathbf{X}) + \epsilon,$$

$$\text{Binary outcome: } Y(a) \sim \text{Bern}(e(\mathbf{X}, a)), \text{ with } e(\mathbf{X}, a) = \{1 + \exp(-20\mathbf{X}'\boldsymbol{\beta}_0 - a\delta(\mathbf{X}))\}^{-1},$$

and we consider two specifications of $\delta(\mathbf{X})$:

- linear: $\delta(\mathbf{X}) = d_1 + c_1\mathbf{X}\boldsymbol{\beta}_1$, and
- nonlinear: $\delta(\mathbf{X}) = d_2 + c_2\mathbf{W}\boldsymbol{\beta}_1$, with $\mathbf{W} = (X_1^2, -X_2^2, |X_3|, X_4X_5, X_5)$,

where $\boldsymbol{\beta}_0 = (1, 1, 1, 1, 1)'$, and $\boldsymbol{\beta}_1 = (2, 4, 6, 2, 4)'$. For continuous outcomes, $(c_1, d_1, c_2, d_2) = (1, 8.15, 2.92, 0)$; for binary outcomes, $(c_1, c_2) = (10, 2, 2, 2)$. The true ATEs, evaluating by the super-population data ($N = 10^8$), are approximately $\tau = 8.15$ for both continuous outcomes and $\tau = 0.08$ for both binary outcomes.

B.2 Additional simulation results of methods performance

In Figures B.2.1–B.2.16, we present full results (Bias, CP% and Power%) of simulation in Section 4 of the main text.

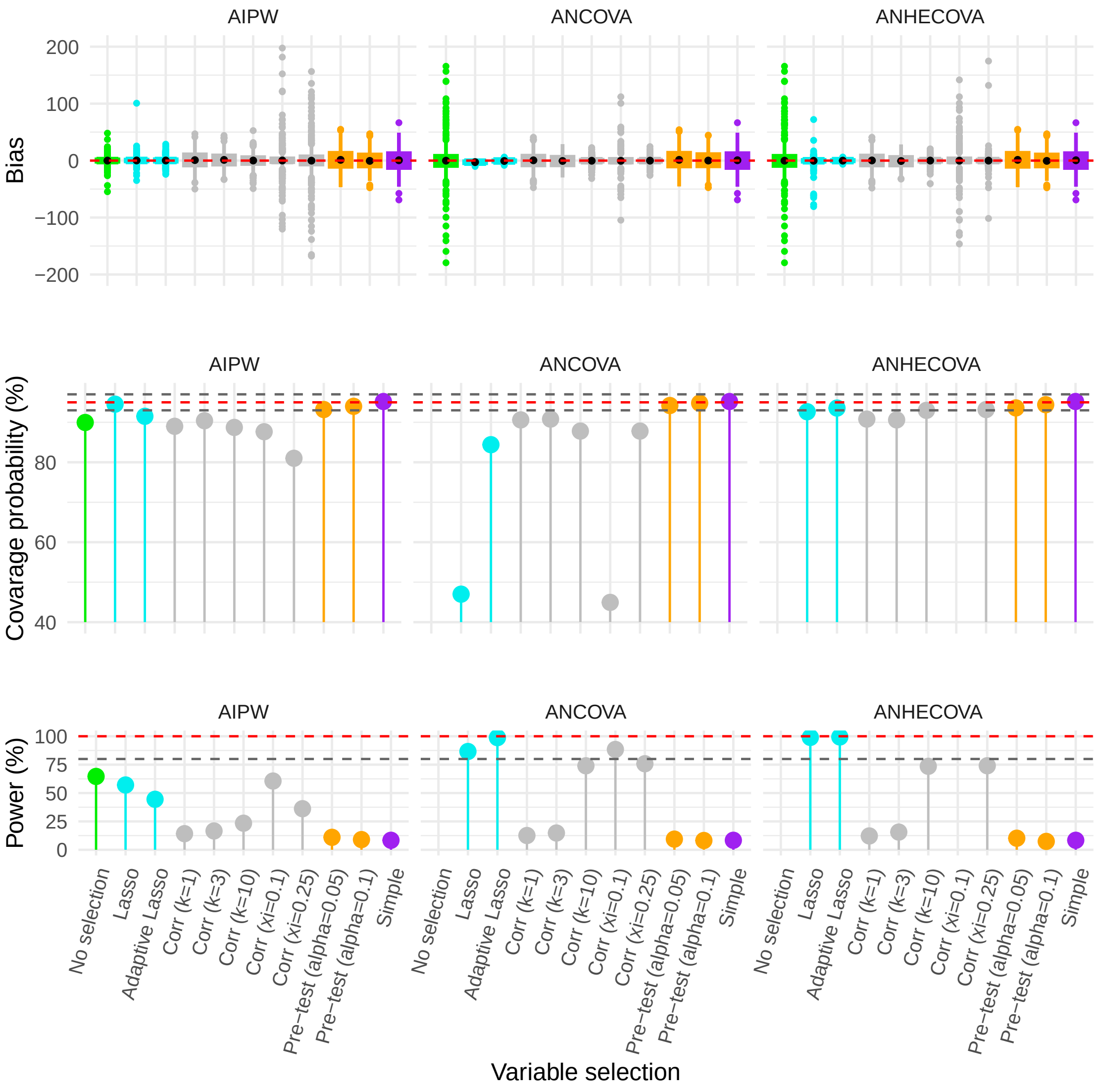

Figure B.2.1: Simulation results under continuous outcome, linear $\delta(\mathbf{X})$ and $N = 40$. In the CP% plots, the red dashed line indicates 95% coverage level, and the two gray dashed lines indicate 93% and 97% coverage levels. In the power plots, the red dashed line indicates 100% power, and the gray dashed line indicates 80% power.

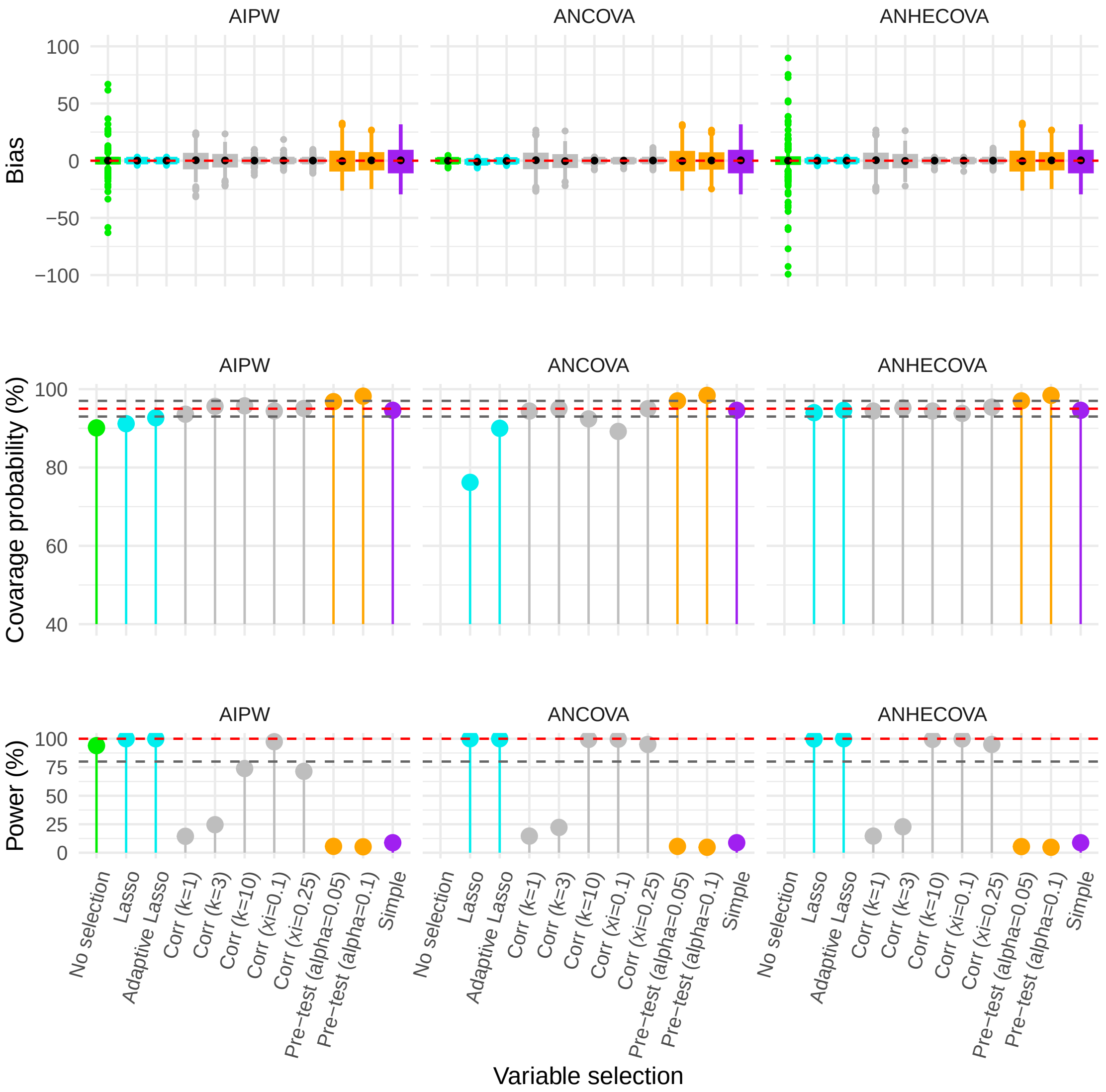

Figure B.2.2: Simulation results under continuous outcome, linear $\delta(\mathbf{X})$ and $N = 100$. In the CP% plots, the red dashed line indicates 95% coverage level, and the two gray dashed lines indicate 93% and 97% coverage levels. In the power plots, the red dashed line indicates 100% power, and the gray dashed line indicates 80% power.

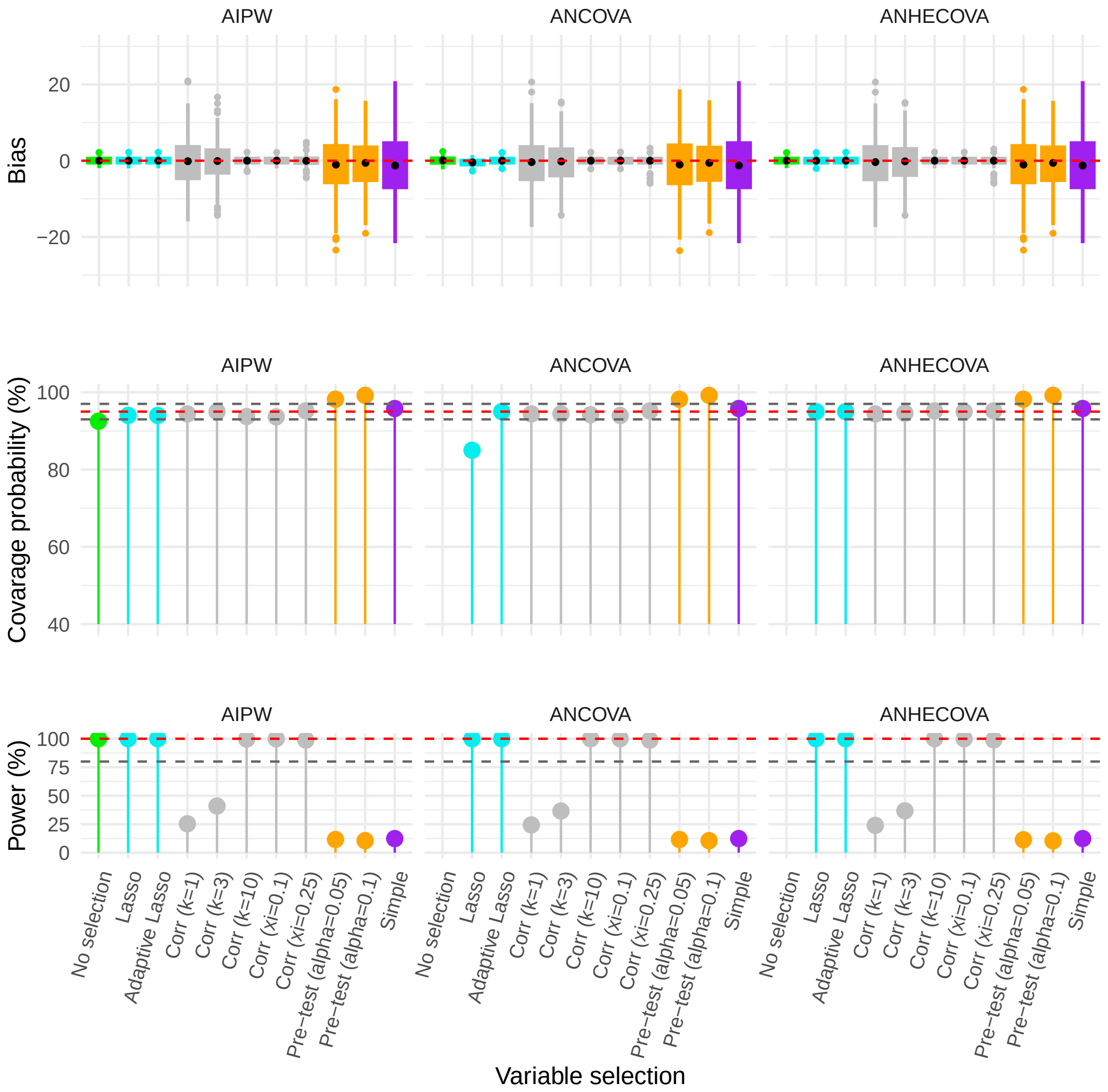

Figure B.2.3: Simulation results under continuous outcome, linear $\delta(\mathbf{X})$ and $N = 200$. In the CP% plots, the red dashed line indicates 95% coverage level, and the two gray dashed lines indicate 93% and 97% coverage levels. In the power plots, the red dashed line indicates 100% power, and the gray dashed line indicates 80% power.

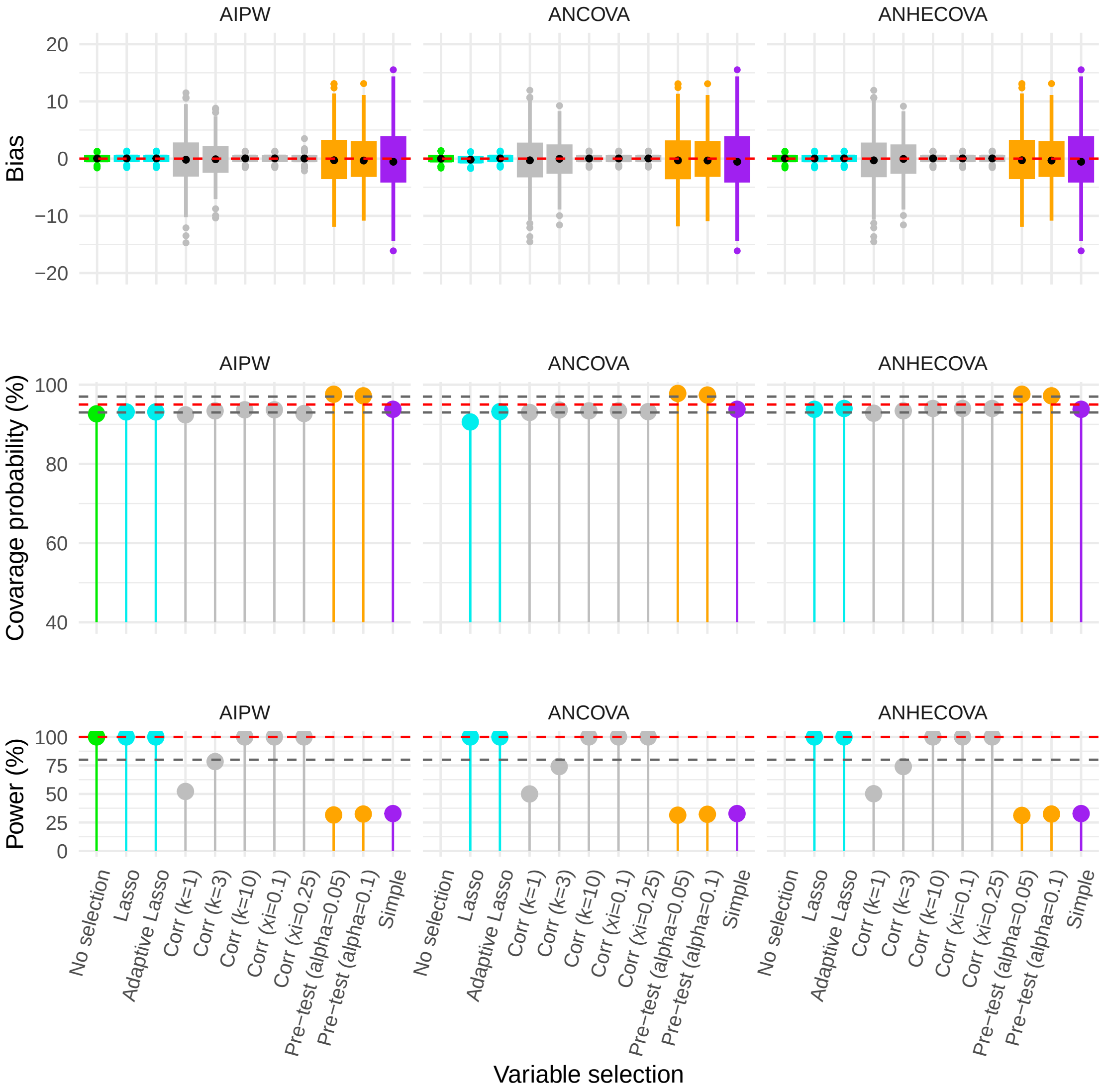

Figure B.2.4: Simulation results under continuous outcome, linear $\delta(\mathbf{X})$ and $N = 500$. In the CP% plots, the red dashed line indicates 95% coverage level, and the two gray dashed lines indicate 93% and 97% coverage levels. In the power plots, the red dashed line indicates 100% power, and the gray dashed line indicates 80% power.

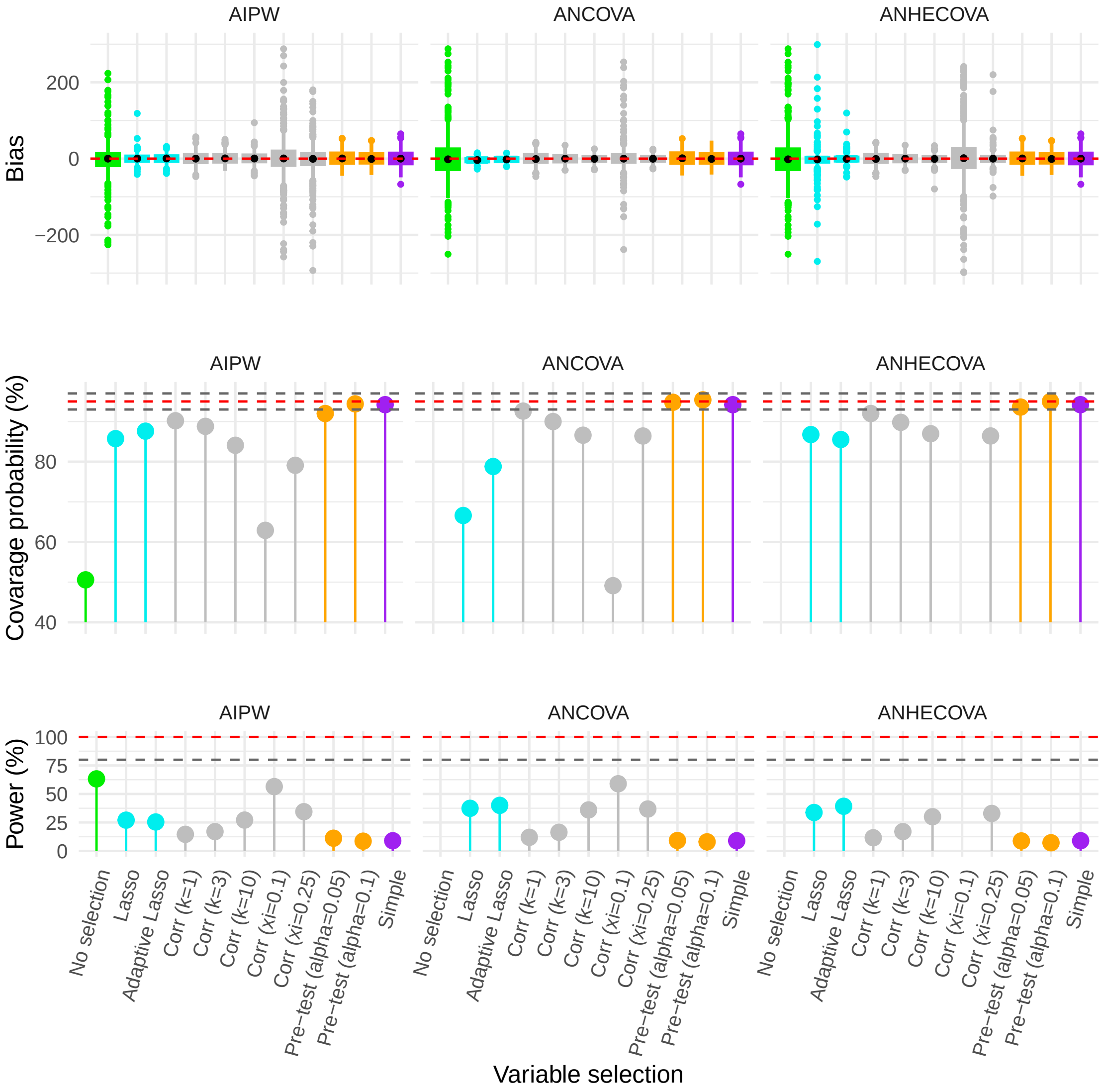

Figure B.2.5: Simulation results under continuous outcome, nonlinear $\delta(\mathbf{X})$ and $N = 40$. In the CP% plots, the red dashed line indicates 95% coverage level, and the two gray dashed lines indicate 93% and 97% coverage levels. In the power plots, the red dashed line indicates 100% power, and the gray dashed line indicates 80% power.

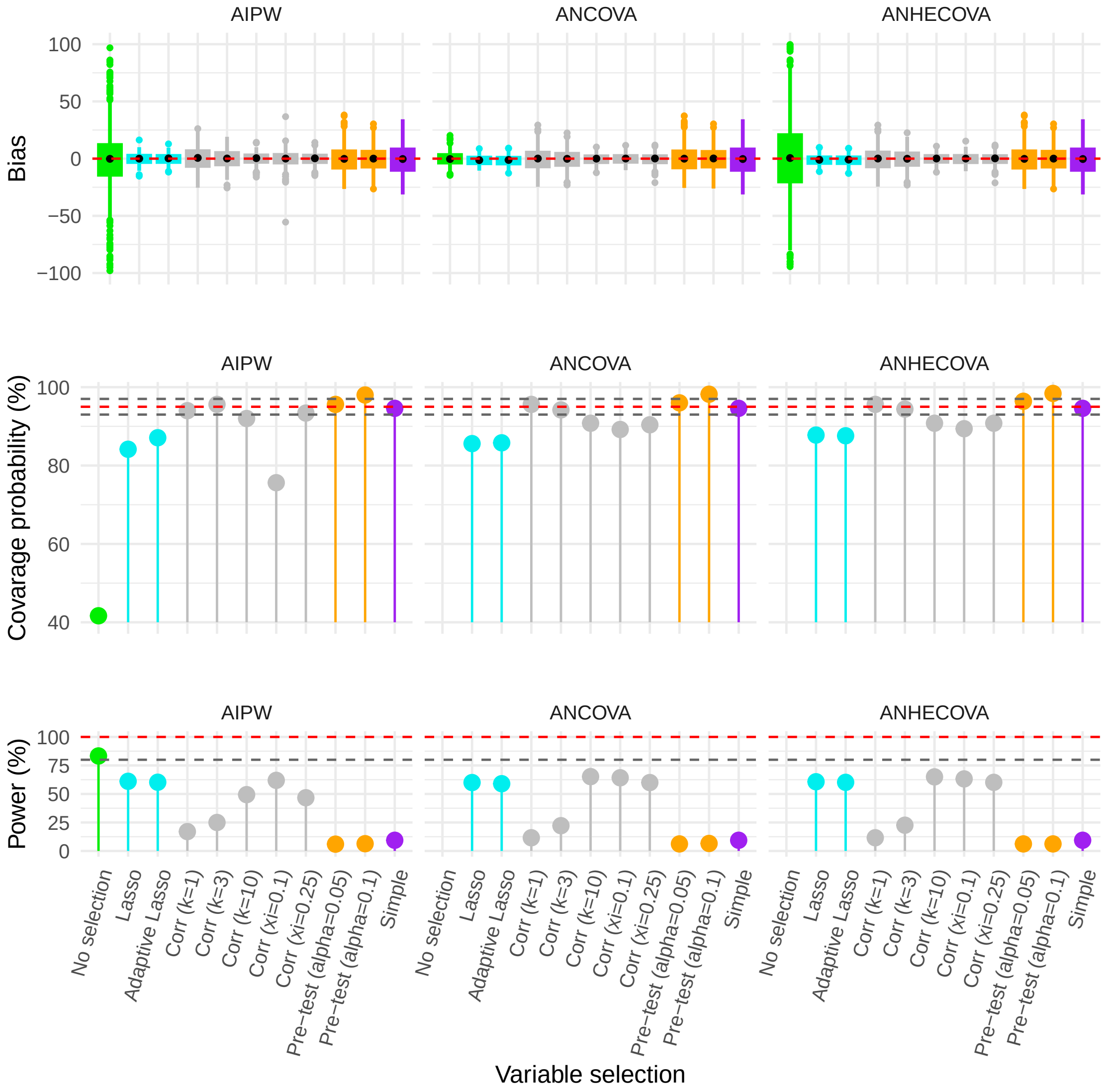

Figure B.2.6: Simulation results under continuous outcome, nonlinear $\delta(\mathbf{X})$ and $N = 100$. In the CP% plots, the red dashed line indicates 95% coverage level, and the two gray dashed lines indicate 93% and 97% coverage levels. In the power plots, the red dashed line indicates 100% power, and the gray dashed line indicates 80% power.

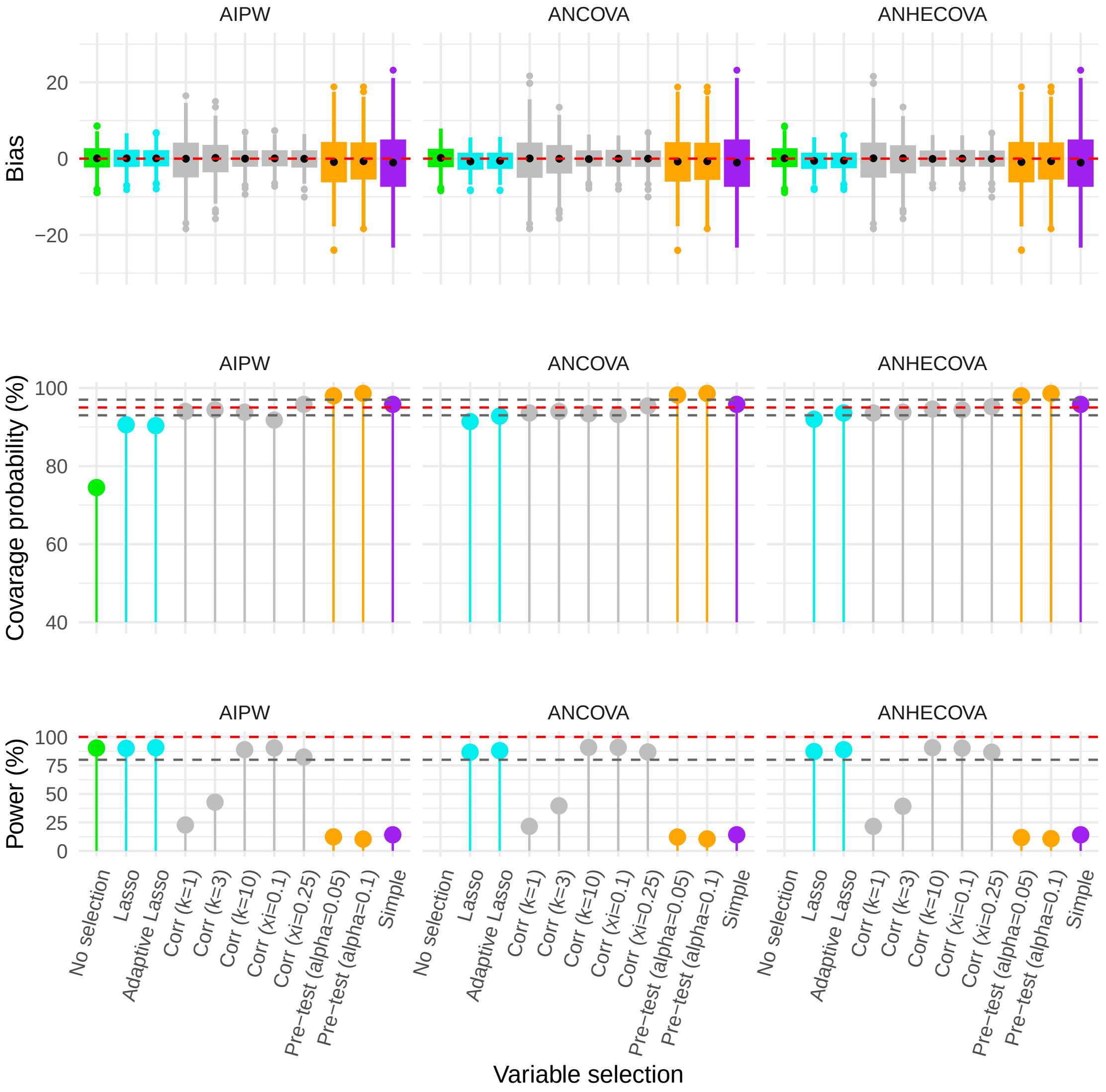

Figure B.2.7: Simulation results under continuous outcome, nonlinear $\delta(\mathbf{X})$ and $N = 200$. In the CP% plots, the red dashed line indicates 95% coverage level, and the two gray dashed lines indicate 93% and 97% coverage levels. In the power plots, the red dashed line indicates 100% power, and the gray dashed line indicates 80% power.

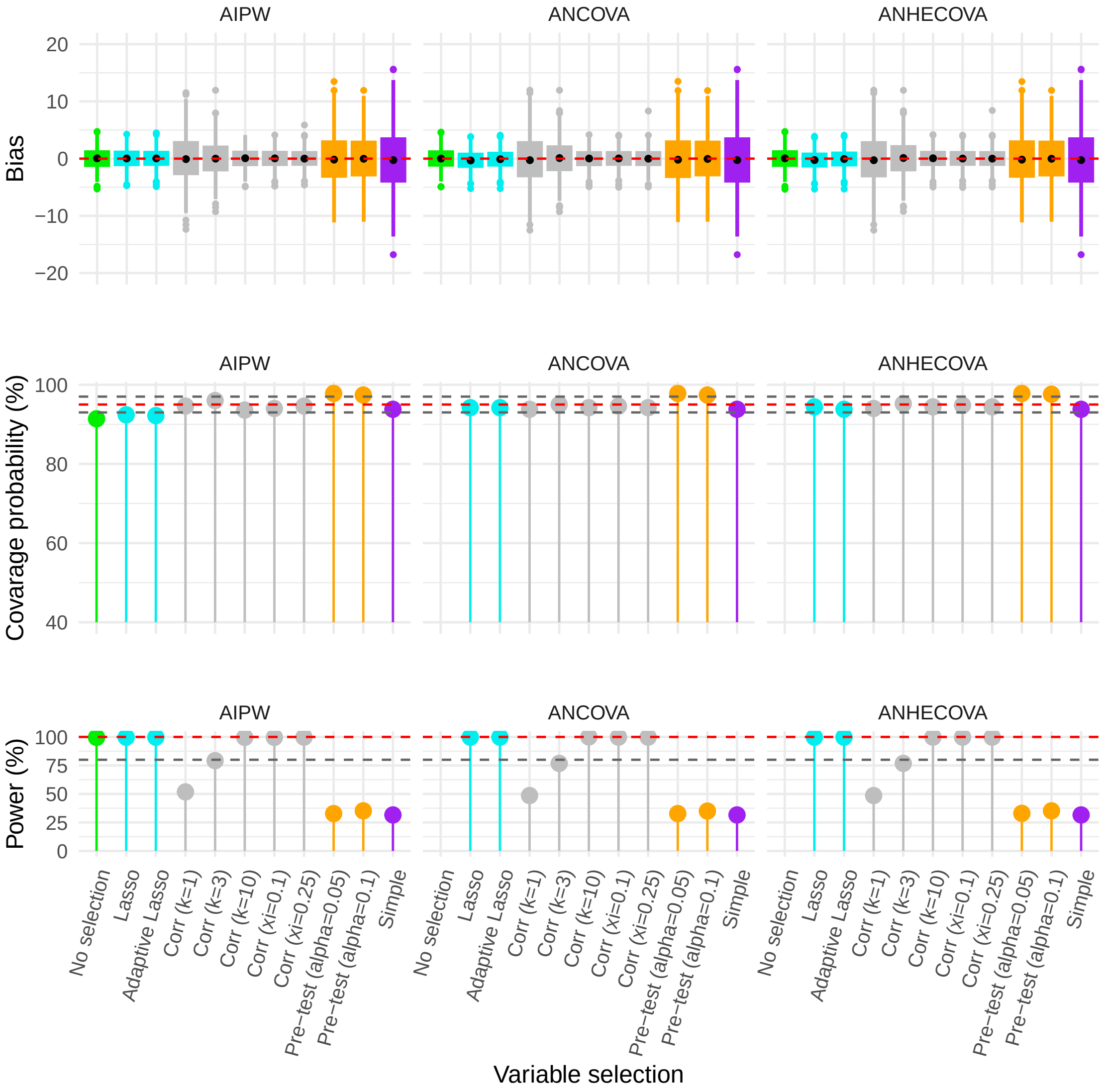

Figure B.2.8: Simulation results under continuous outcome, nonlinear $\delta(\mathbf{X})$ and $N = 500$. In the CP% plots, the red dashed line indicates 95% coverage level, and the two gray dashed lines indicate 93% and 97% coverage levels. In the power plots, the red dashed line indicates 100% power, and the gray dashed line indicates 80% power.

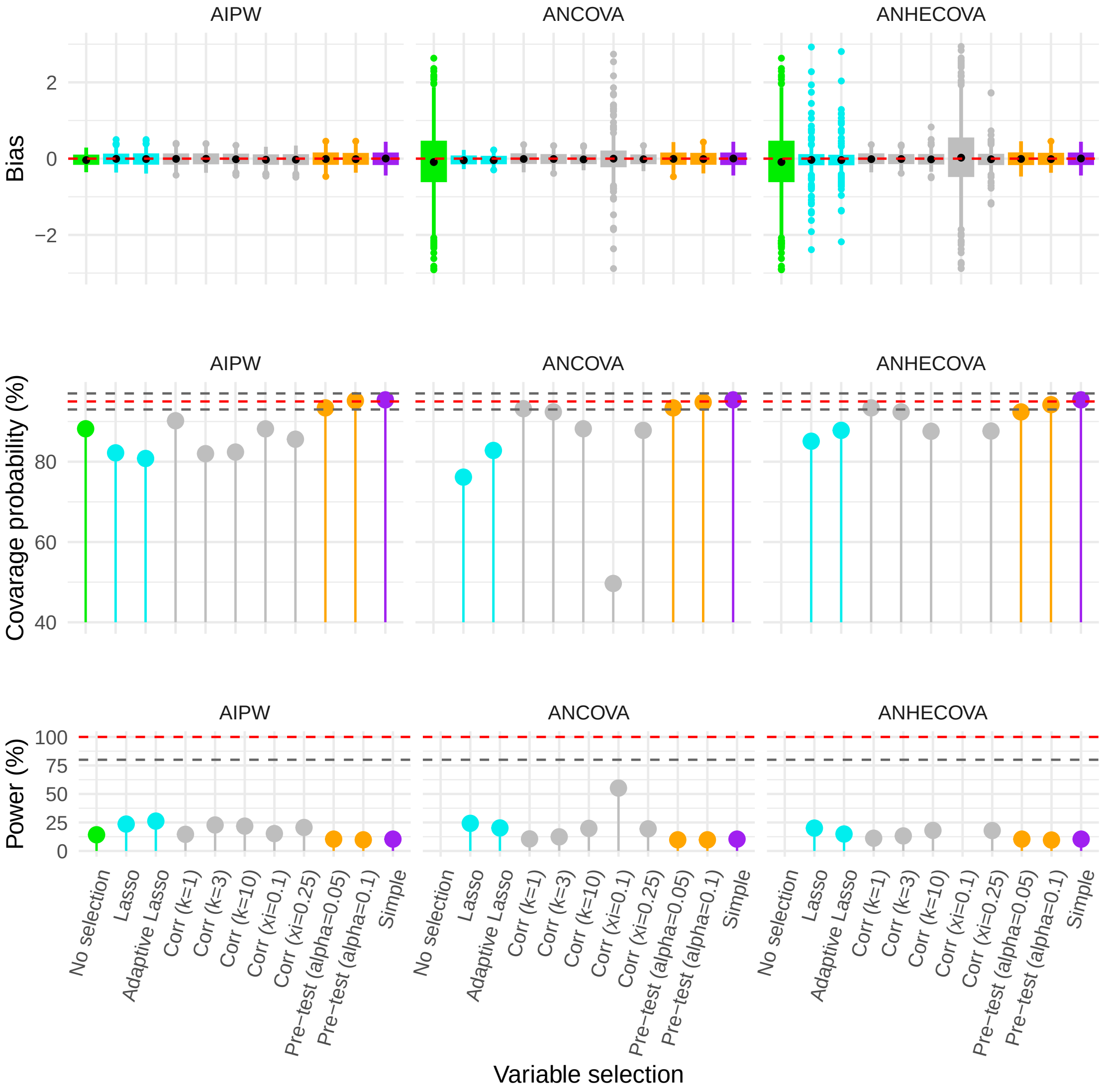

Figure B.2.9: Simulation results under binary outcome, linear $\delta(\mathbf{X})$ and $N = 40$. In the CP% plots, the red dashed line indicates 95% coverage level, and the two gray dashed lines indicate 93% and 97% coverage levels. In the power plots, the red dashed line indicates 100% power, and the gray dashed line indicates 80% power.

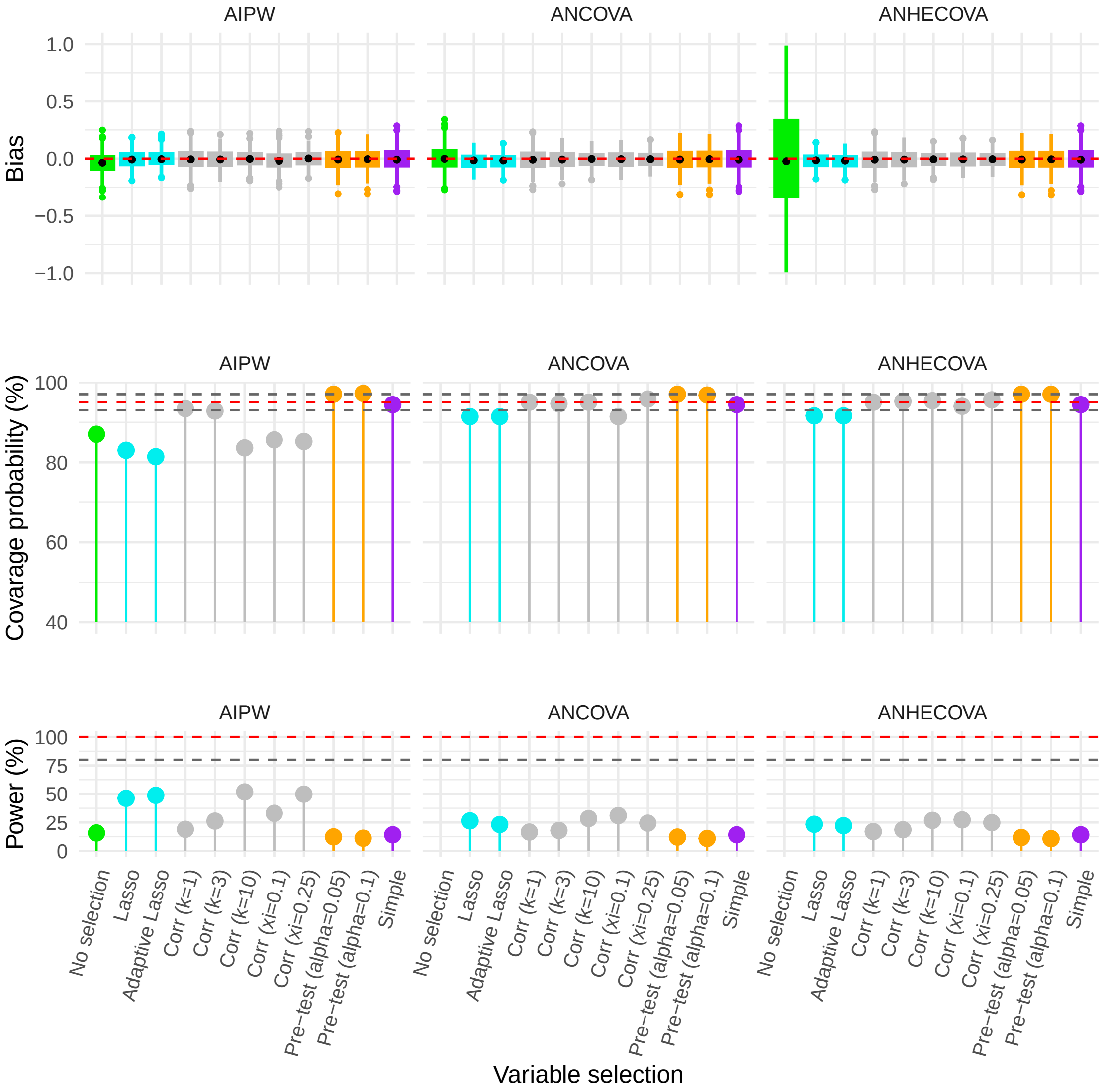

Figure B.2.10: Simulation results under binary outcome, linear $\delta(\mathbf{X})$ and $N = 100$. In the CP% plots, the red dashed line indicates 95% coverage level, and the two gray dashed lines indicate 93% and 97% coverage levels. In the power plots, the red dashed line indicates 100% power, and the gray dashed line indicates 80% power.

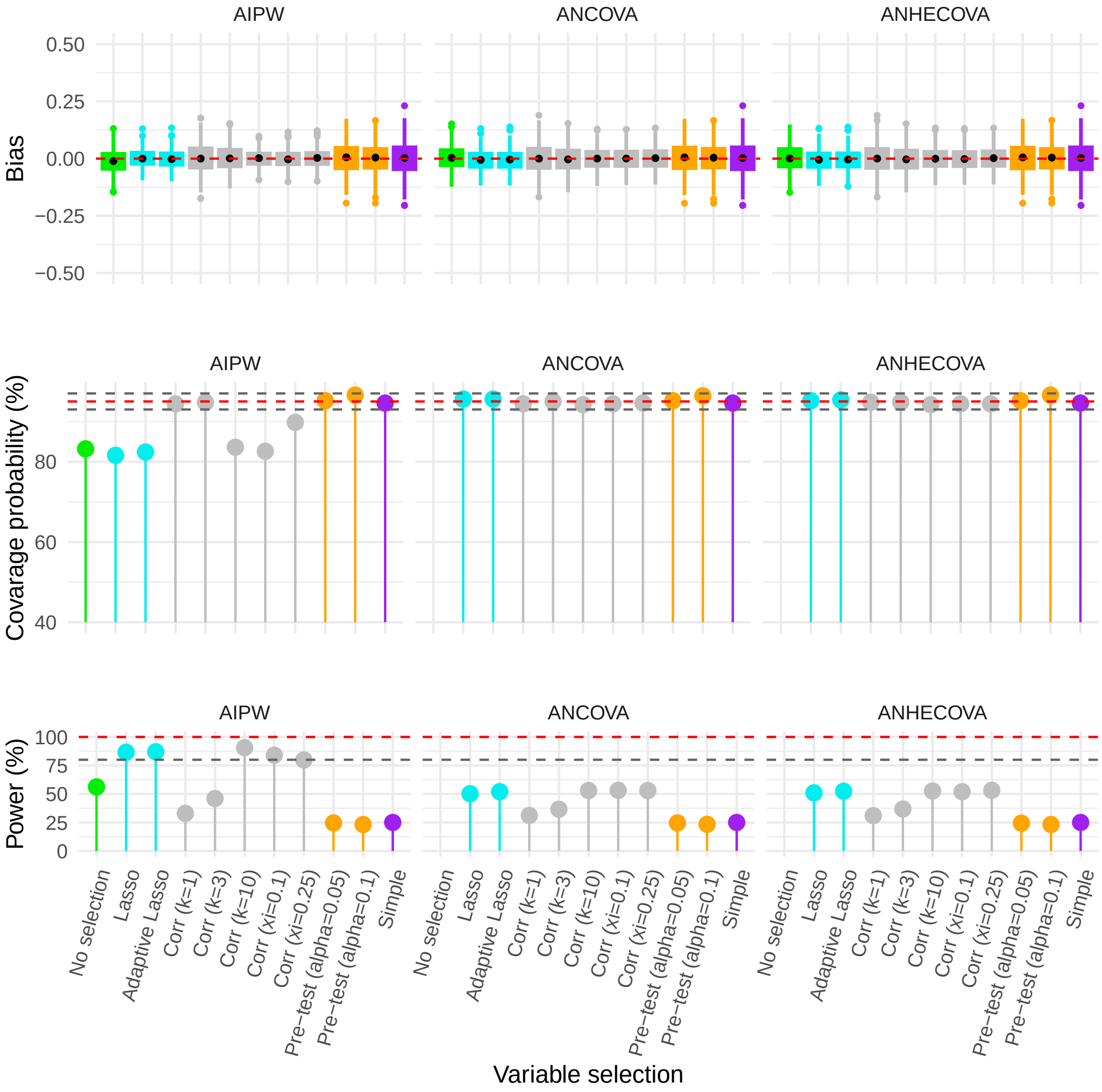

Figure B.2.11: Simulation results under binary outcome, linear $\delta(\mathbf{X})$ and $N = 200$. In the CP% plots, the red dashed line indicates 95% coverage level, and the two gray dashed lines indicate 93% and 97% coverage levels. In the power plots, the red dashed line indicates 100% power, and the gray dashed line indicates 80% power.

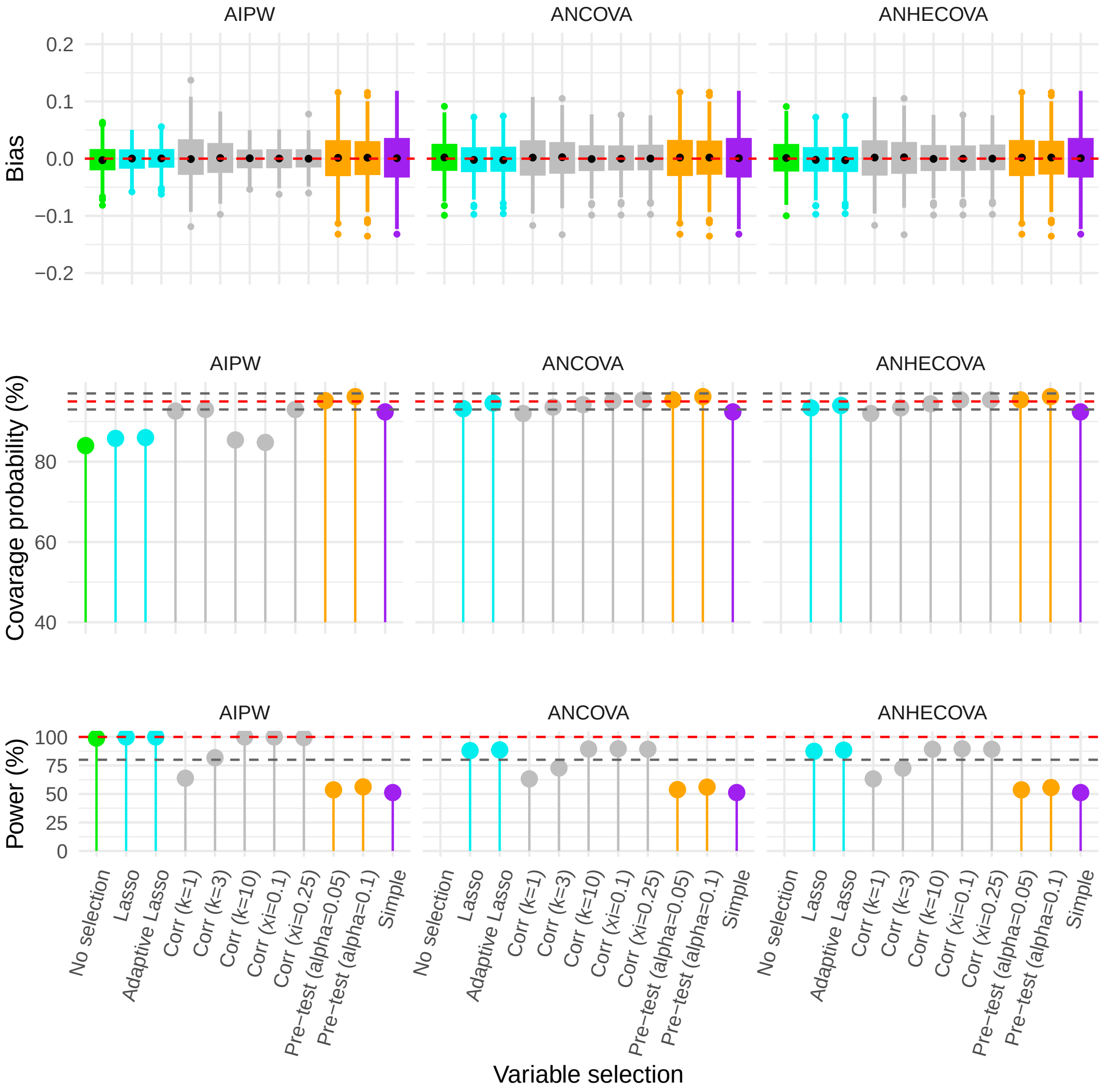

Figure B.2.12: Simulation results under binary outcome, linear $\delta(\mathbf{X})$ and $N = 500$. In the CP% plots, the red dashed line indicates 95% coverage level, and the two gray dashed lines indicate 93% and 97% coverage levels. In the power plots, the red dashed line indicates 100% power, and the gray dashed line indicates 80% power.

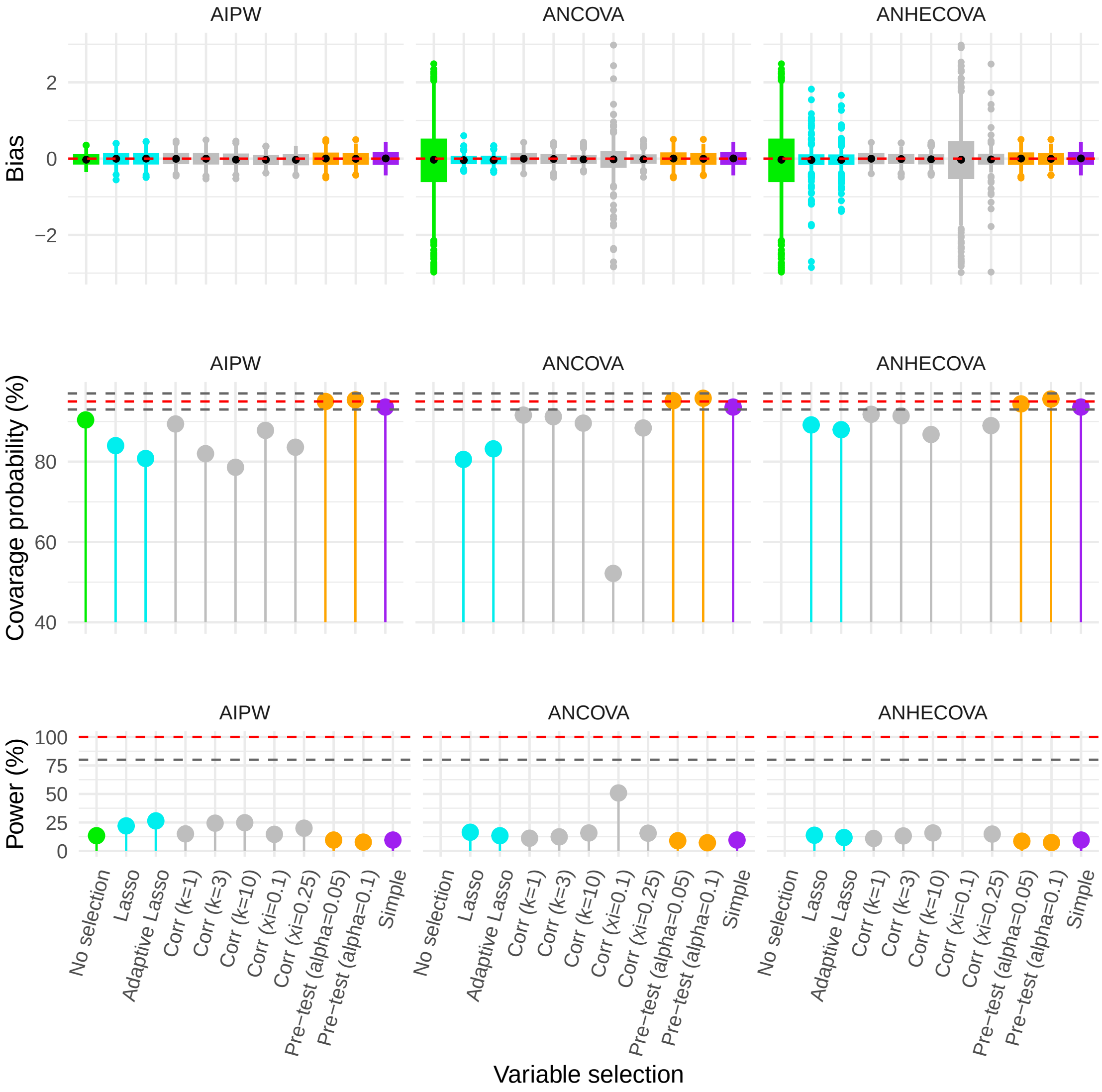

Figure B.2.13: Simulation results under binary outcome, nonlinear $\delta(\mathbf{X})$ and $N = 40$. In the CP% plots, the red dashed line indicates 95% coverage level, and the two gray dashed lines indicate 93% and 97% coverage levels. In the power plots, the red dashed line indicates 100% power, and the gray dashed line indicates 80% power.

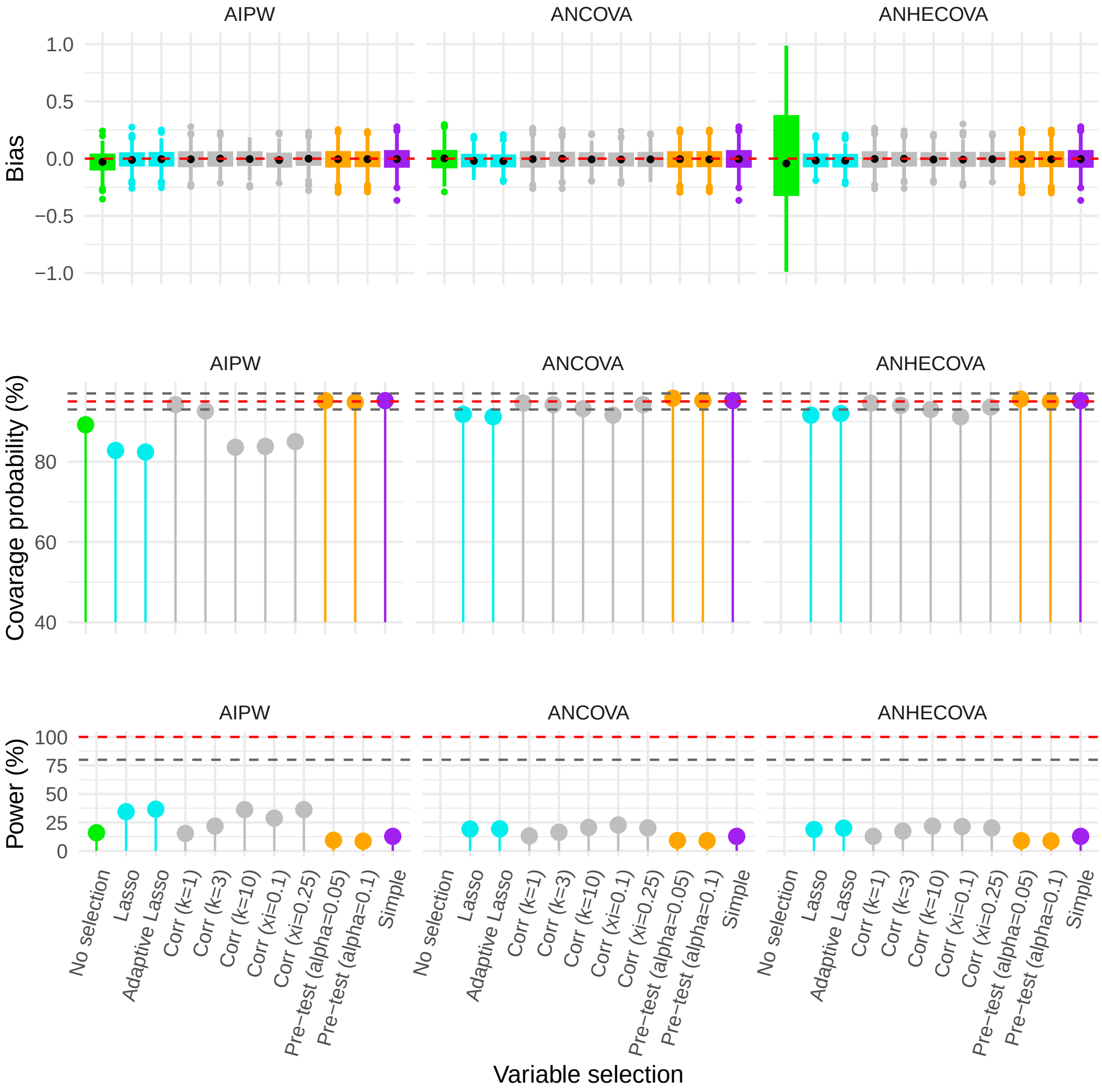

Figure B.2.14: Simulation results under binary outcome, nonlinear $\delta(\mathbf{X})$ and $N = 100$. In the CP% plots, the red dashed line indicates 95% coverage level, and the two gray dashed lines indicate 93% and 97% coverage levels. In the power plots, the red dashed line indicates 100% power, and the gray dashed line indicates 80% power.

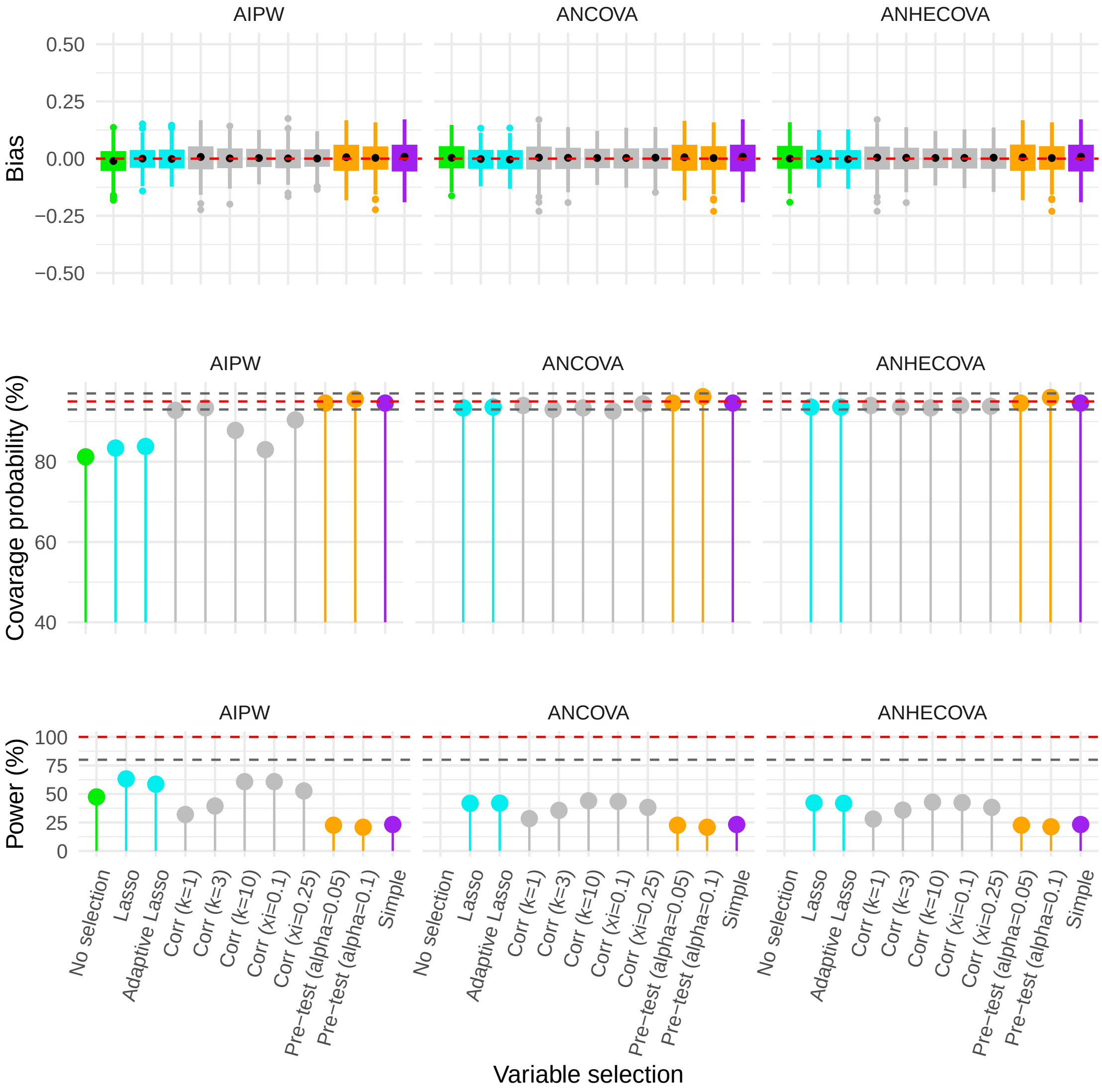

Figure B.2.15: Simulation results under binary outcome, nonlinear $\delta(\mathbf{X})$ and $N = 200$. In the CP% plots, the red dashed line indicates 95% coverage level, and the two gray dashed lines indicate 93% and 97% coverage levels. In the power plots, the red dashed line indicates 100% power, and the gray dashed line indicates 80% power.

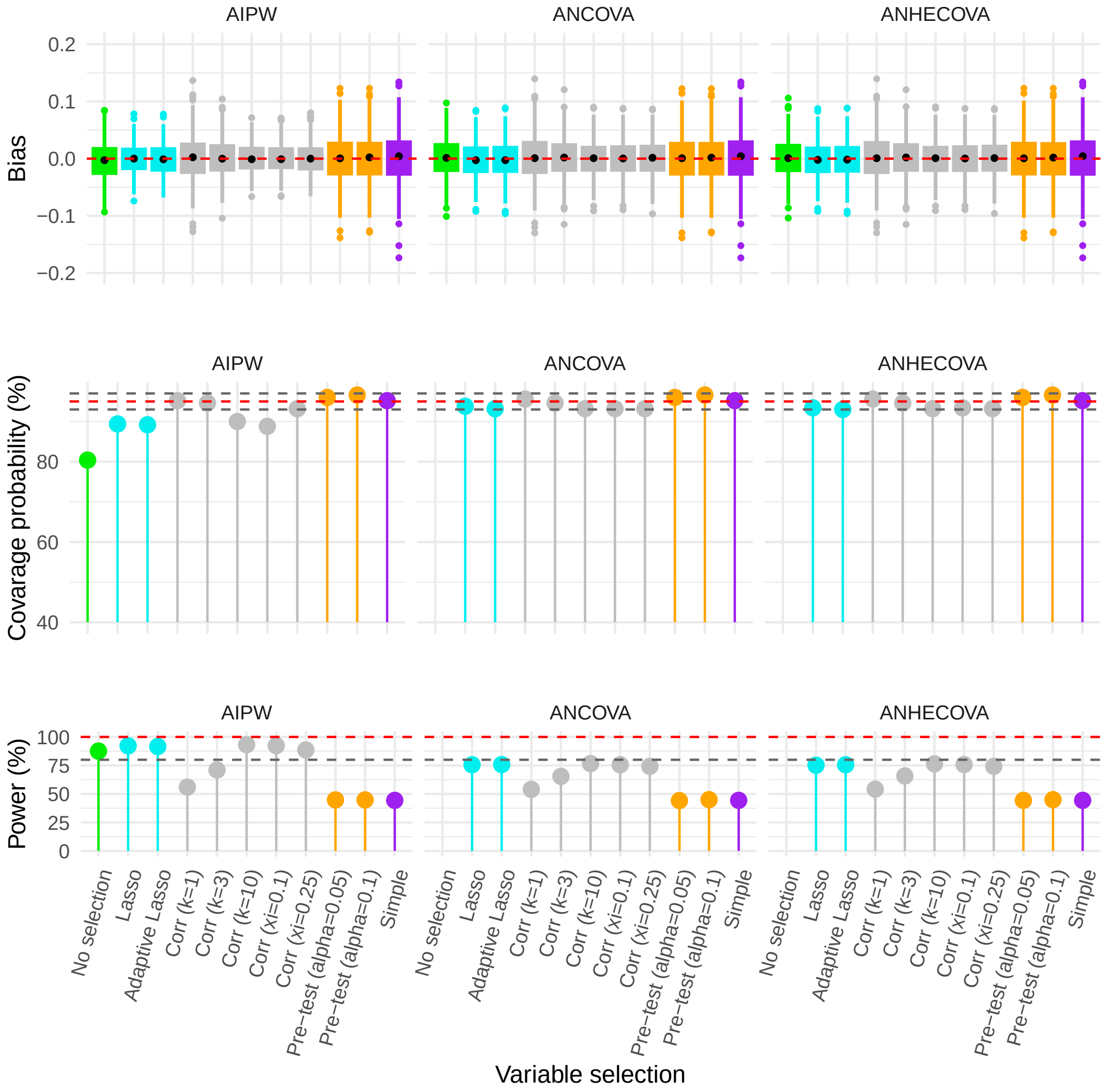

Figure B.2.16: Simulation results under binary outcome, nonlinear $\delta(\mathbf{X})$ and $N = 500$. In the CP% plots, the red dashed line indicates 95% coverage level, and the two gray dashed lines indicate 93% and 97% coverage levels. In the power plots, the red dashed line indicates 100% power, and the gray dashed line indicates 80% power.

B.3 Additional simulation results of estimate distributions

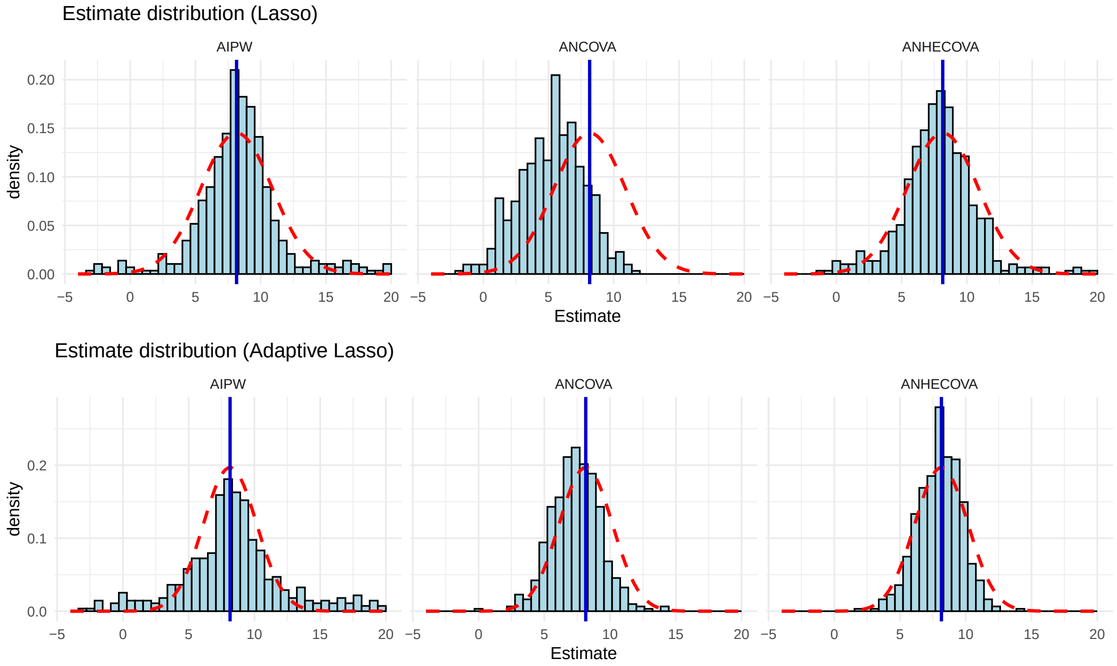

Figure B.3.1: Simulation results for estimation distributions by Lasso and adaptive Lasso variable selections, under continuous outcome, linear $\delta(\mathbf{X})$ and $N = 40$. The red dashed curves are the theoretical normal densities curves, and the blue lines indicate the true ATE.

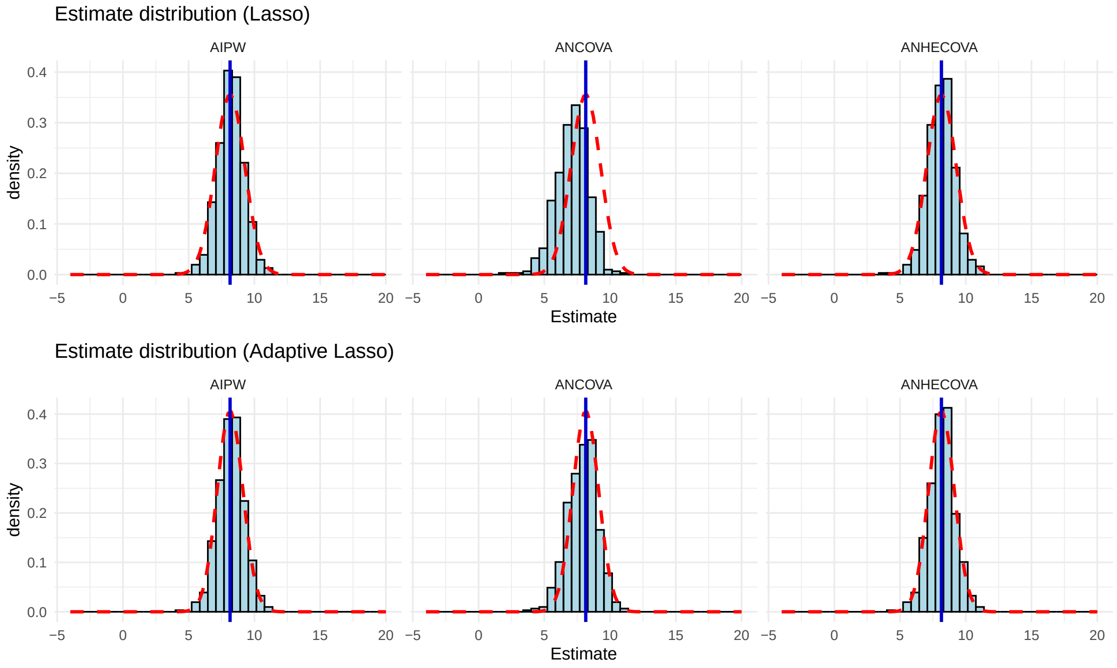

Figure B.3.2: Simulation results for estimation distributions by Lasso and adaptive Lasso variable selections, under continuous outcome, linear $\delta(\mathbf{X})$ and $N = 100$. The red dashed curves are the theoretical normal densities curves, and the blue lines indicate the true ATE.

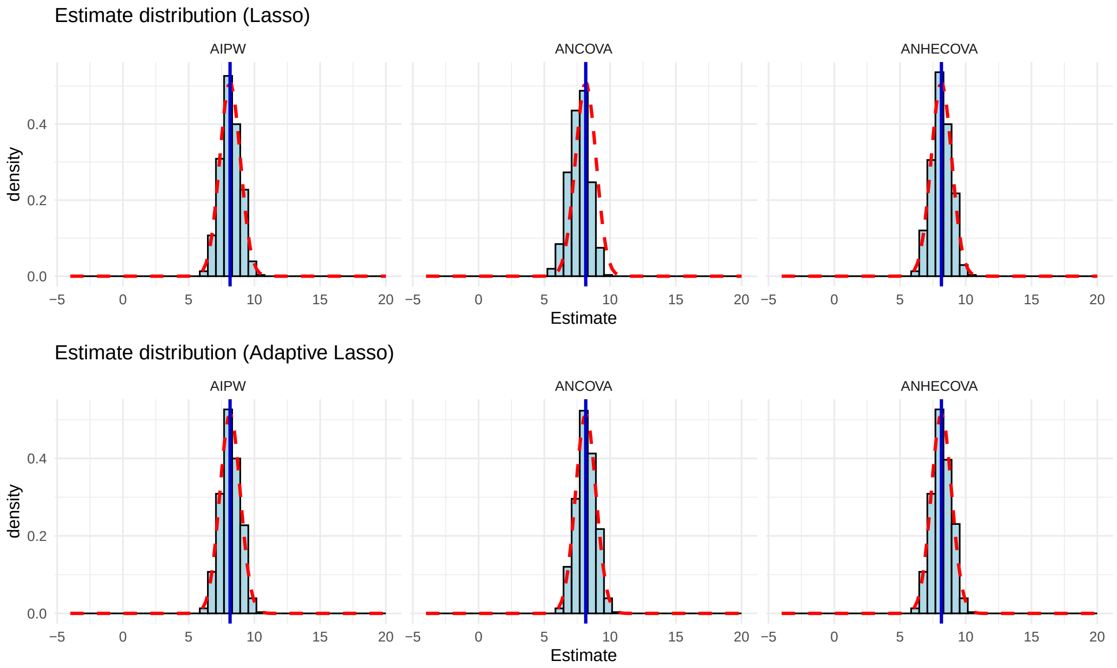

Figure B.3.3: Simulation results for estimation distributions by Lasso and adaptive Lasso variable selections, under continuous outcome, linear $\delta(\mathbf{X})$ and $N = 200$. The red dashed curves are the theoretical normal densities curves, and the blue lines indicate the true ATE.

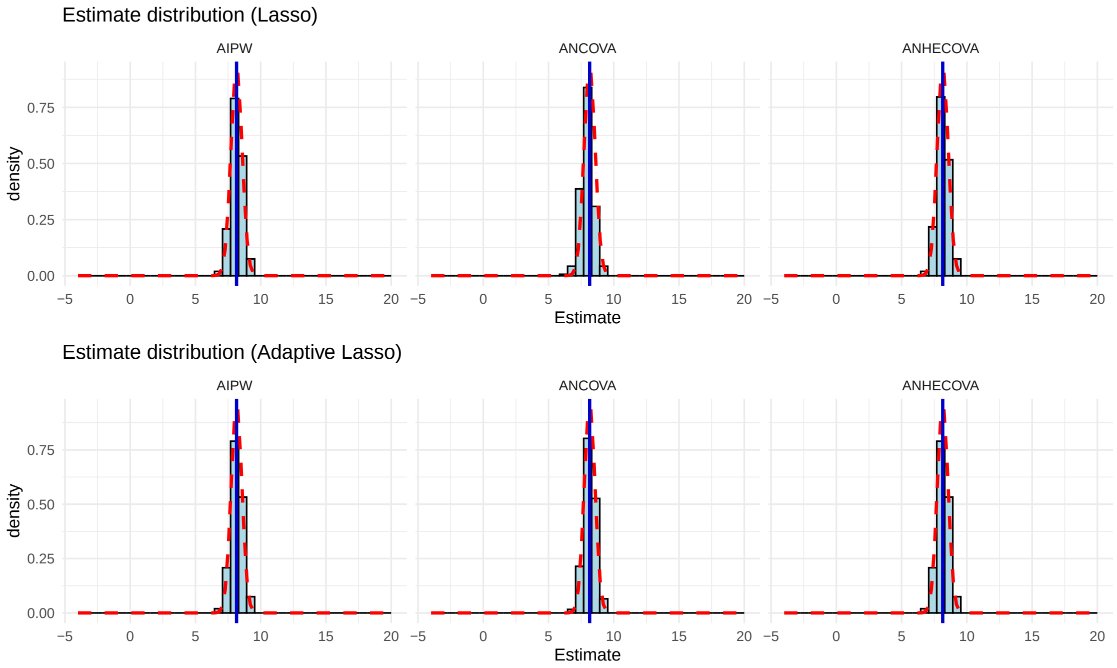

Figure B.3.4: Simulation results for estimation distributions by Lasso and adaptive Lasso variable selections, under continuous outcome, linear $\delta(\mathbf{X})$ and $N = 500$. The red dashed curves are the theoretical normal densities curves, and the blue lines indicate the true ATE.

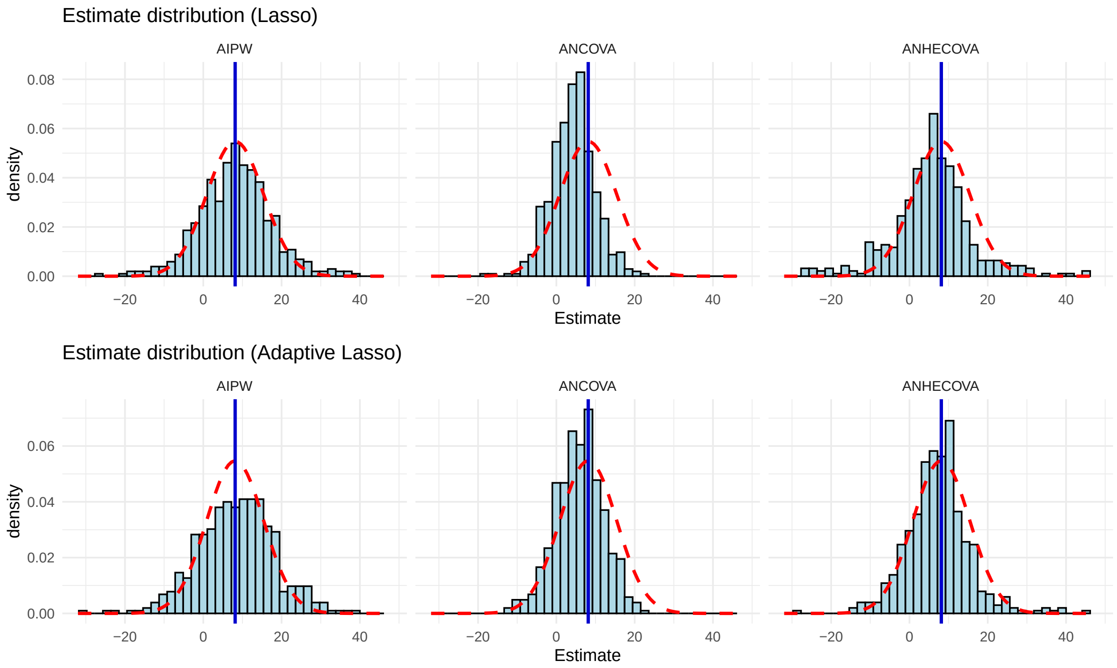

Figure B.3.5: Simulation results for estimation distributions by Lasso and adaptive Lasso variable selections, under continuous outcome, nonlinear $\delta(\mathbf{X})$ and $N = 40$. The red dashed curves are the theoretical normal densities curves, and the blue lines indicate the true ATE.

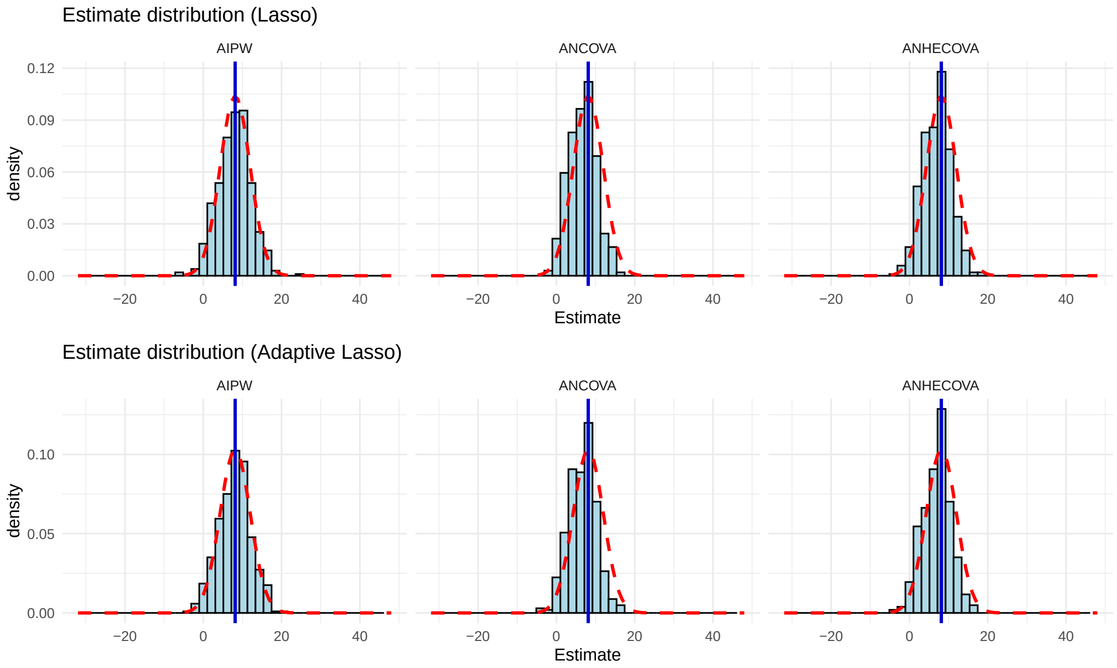

Figure B.3.6: Simulation results for estimation distributions by Lasso and adaptive Lasso variable selections, under continuous outcome, nonlinear $\delta(\mathbf{X})$ and $N = 100$. The red dashed curves are the theoretical normal densities curves, and the blue lines indicate the true ATE.

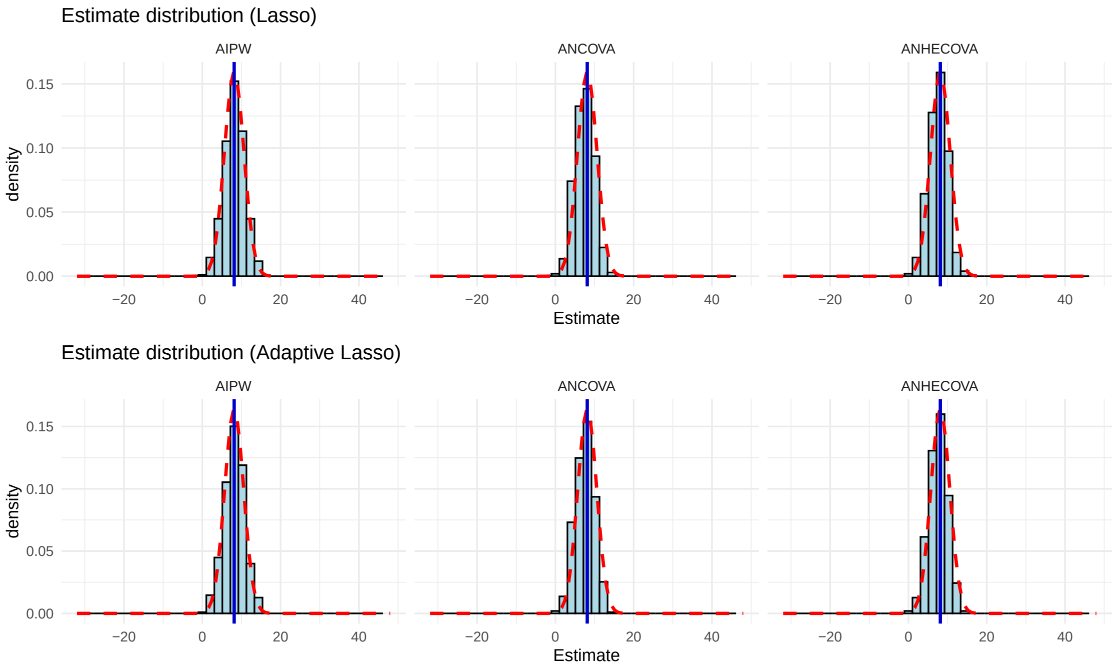

Figure B.3.7: Simulation results for estimation distributions by Lasso and adaptive Lasso variable selections, under continuous outcome, nonlinear $\delta(\mathbf{X})$ and $N = 200$. The red dashed curves are the theoretical normal densities curves, and the blue lines indicate the true ATE.

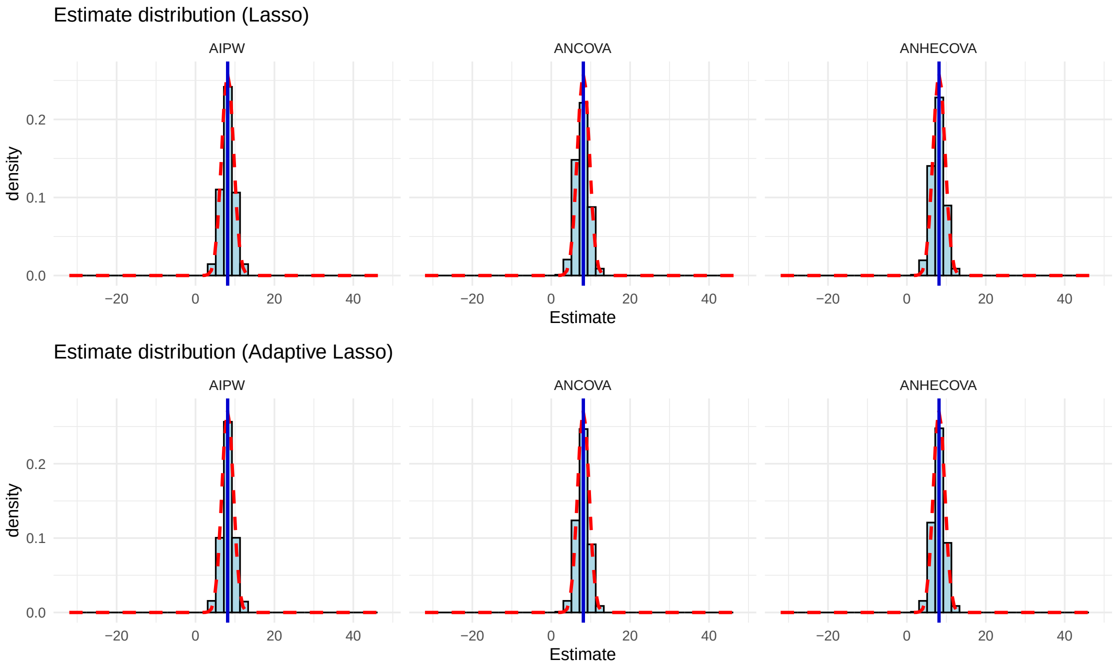

Figure B.3.8: Simulation results for estimation distributions by Lasso and adaptive Lasso variable selections, under continuous outcome, nonlinear $\delta(\mathbf{X})$ and $N = 500$. The red dashed curves are the theoretical normal densities curves, and the blue lines indicate the true ATE.

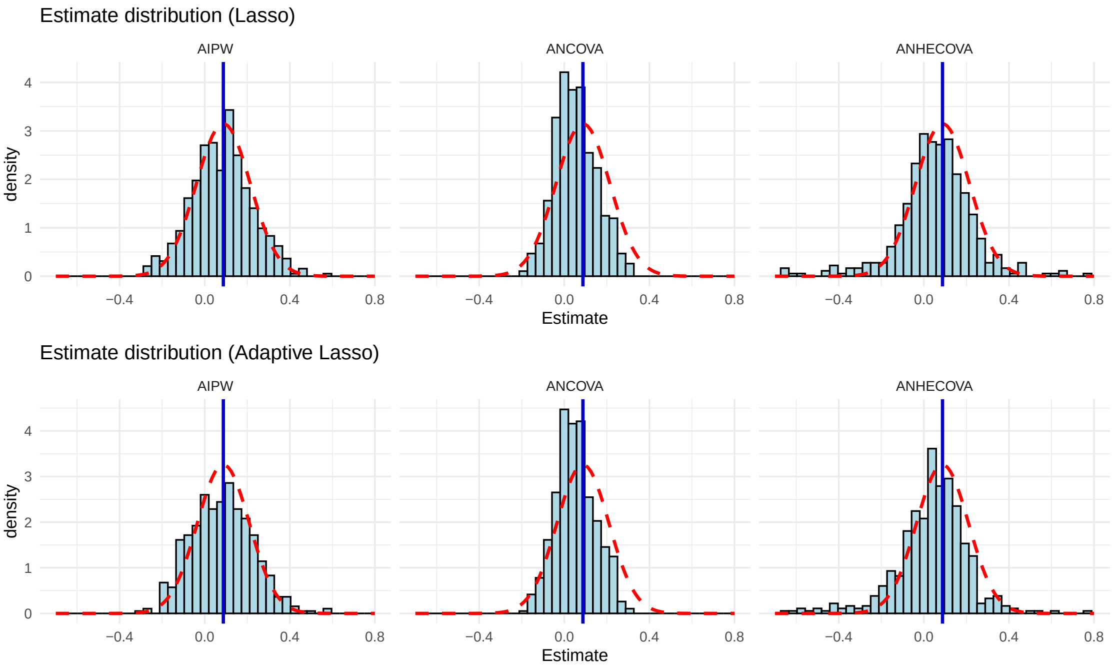

Figure B.3.9: Simulation results for estimation distributions by Lasso and adaptive Lasso variable selections, under binary outcome, linear $\delta(\mathbf{X})$ and $N = 40$. The red dashed curves are the theoretical normal densities curves, and the blue lines indicate the true ATE.

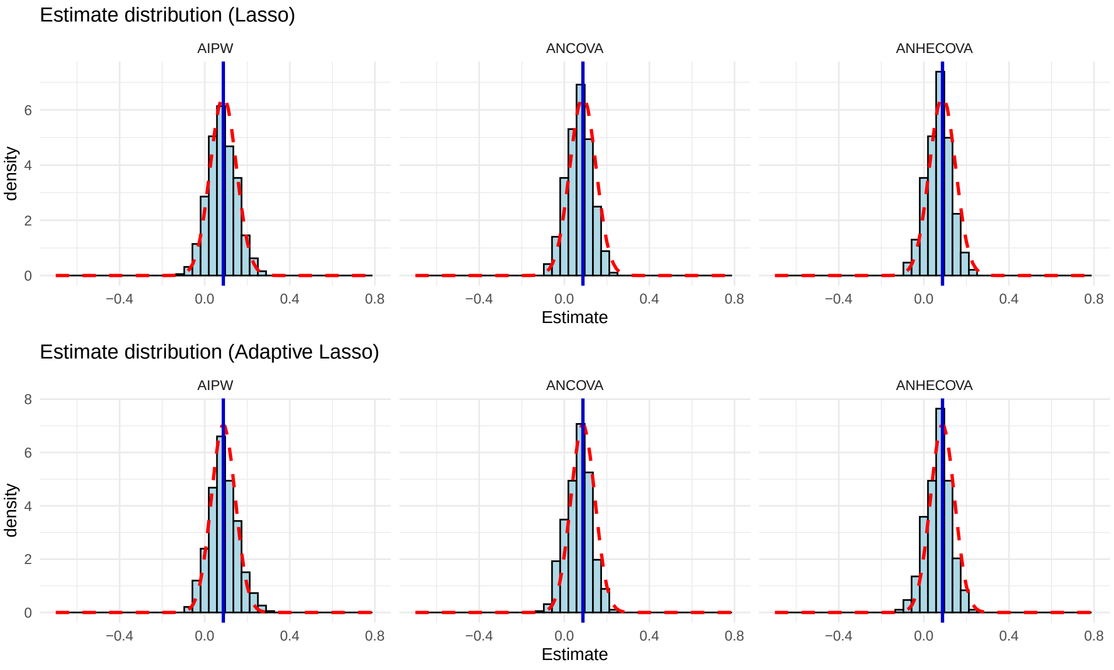

Figure B.3.10: Simulation results for estimation distributions by Lasso and adaptive Lasso variable selections, under binary outcome, linear $\delta(\mathbf{X})$ and $N = 100$. The red dashed curves are the theoretical normal densities curves, and the blue lines indicate the true ATE.

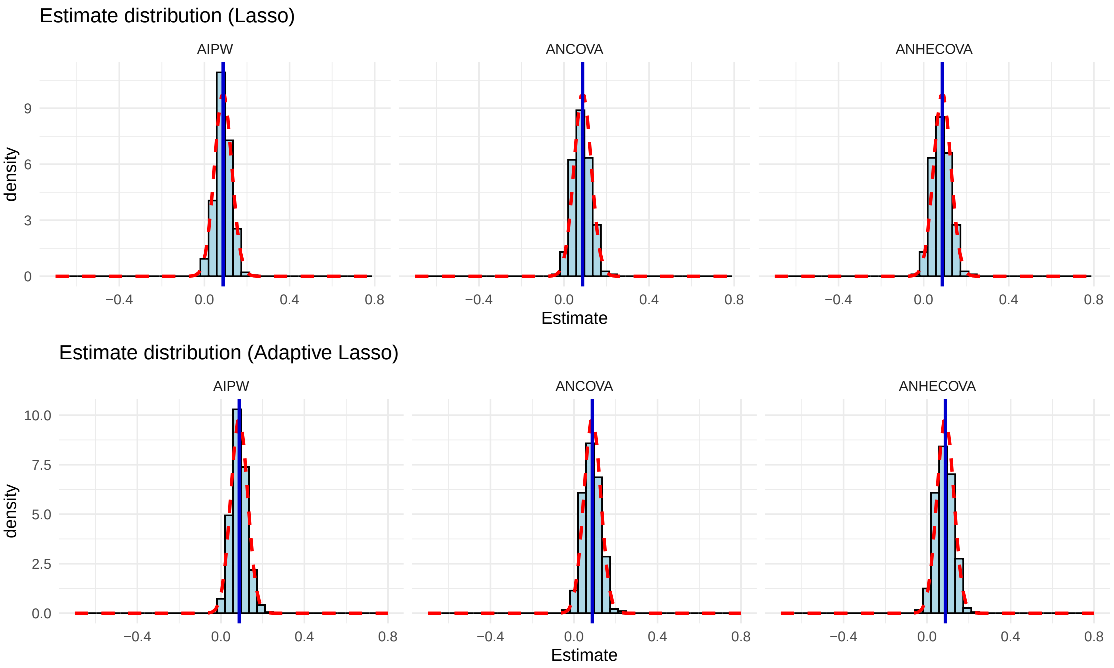

Figure B.3.11: Simulation results for estimation distributions by Lasso and adaptive Lasso variable selections, under binary outcome, linear $\delta(\mathbf{X})$ and $N = 200$. The red dashed curves are the theoretical normal densities curves, and the blue lines indicate the true ATE.

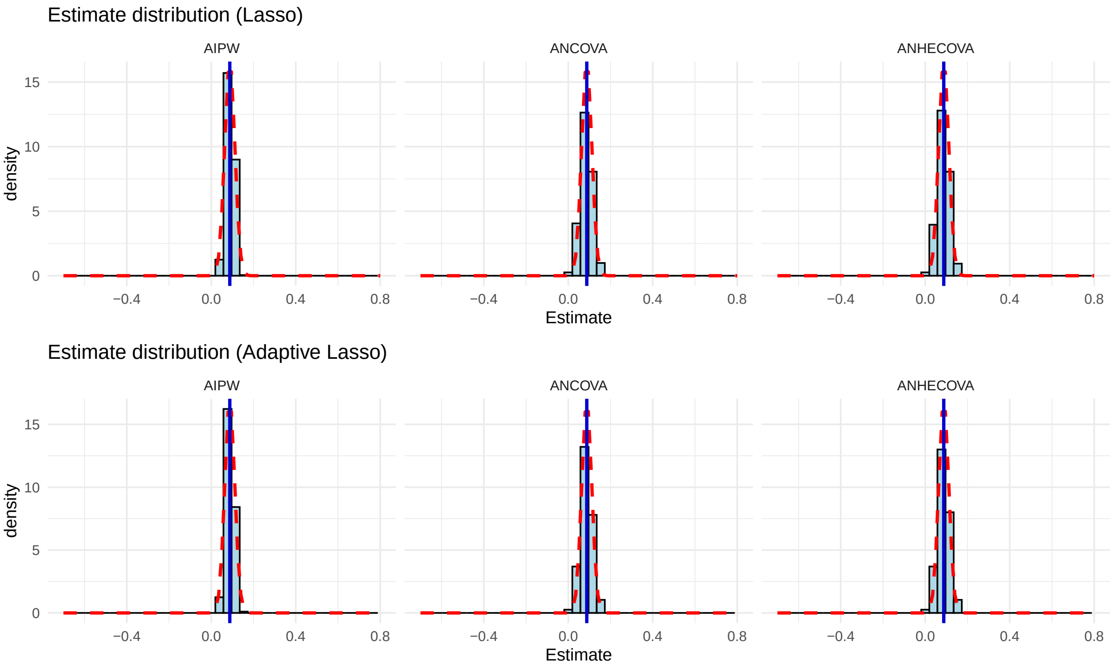

Figure B.3.12: Simulation results for estimation distributions by Lasso and adaptive Lasso variable selections, under binary outcome, linear $\delta(\mathbf{X})$ and $N = 500$. The red dashed curves are the theoretical normal densities curves, and the blue lines indicate the true ATE.

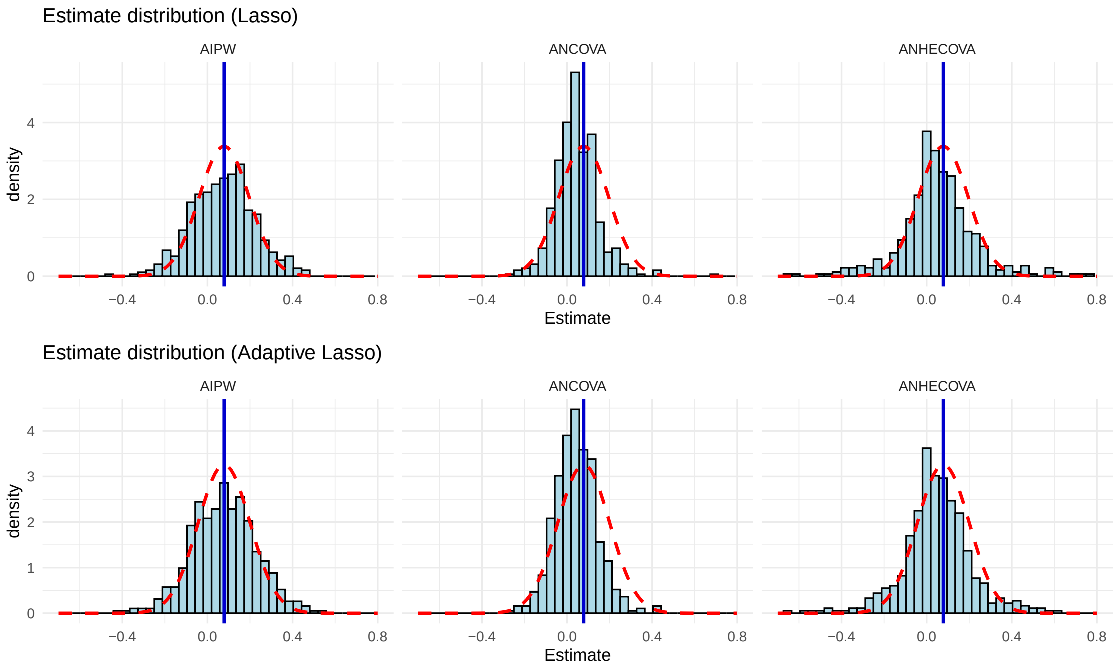

Figure B.3.13: Simulation results for estimation distributions by Lasso and adaptive Lasso variable selections, under binary outcome, nonlinear $\delta(\mathbf{X})$ and $N = 40$. The red dashed curves are the theoretical normal densities curves, and the blue lines indicate the true ATE.

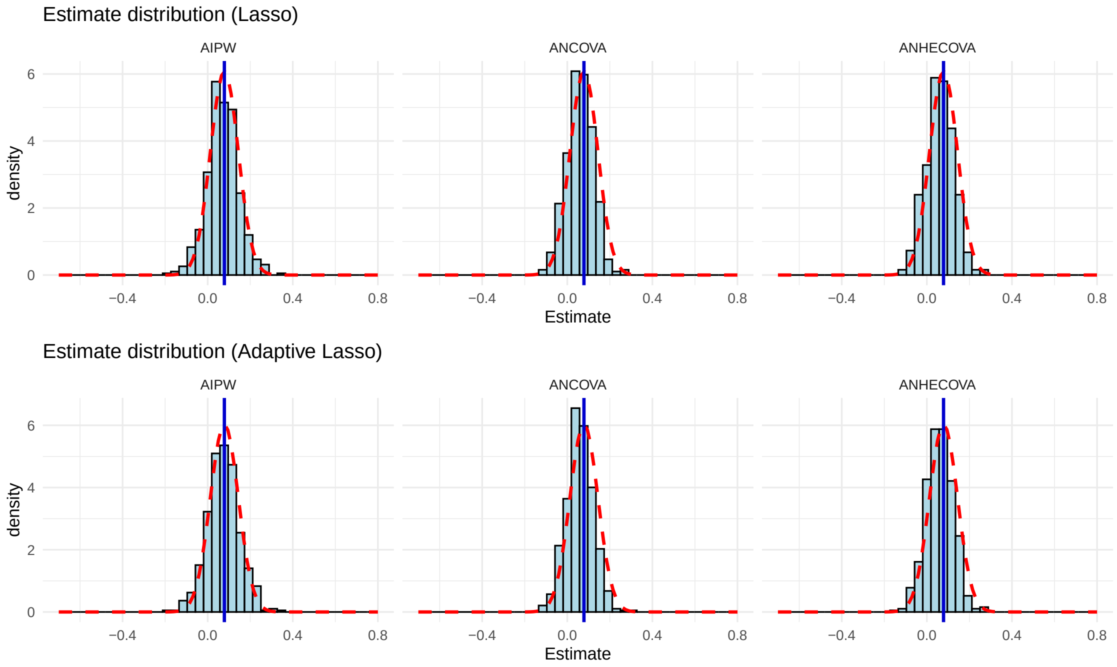

Figure B.3.14: Simulation results for estimation distributions by Lasso and adaptive Lasso variable selections, under binary outcome, nonlinear $\delta(\mathbf{X})$ and $N = 100$. The red dashed curves are the theoretical normal densities curves, and the blue lines indicate the true ATE.

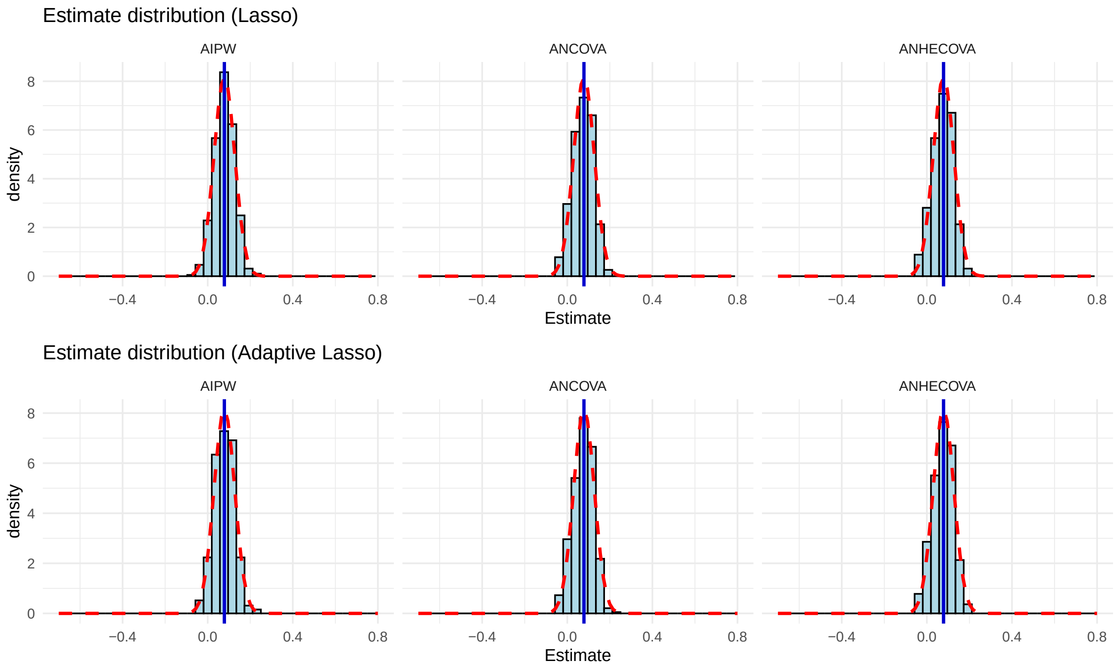

Figure B.3.15: Simulation results for estimation distributions by Lasso and adaptive Lasso variable selections, under binary outcome, nonlinear $\delta(\mathbf{X})$ and $N = 200$. The red dashed curves are the theoretical normal densities curves, and the blue lines indicate the true ATE.

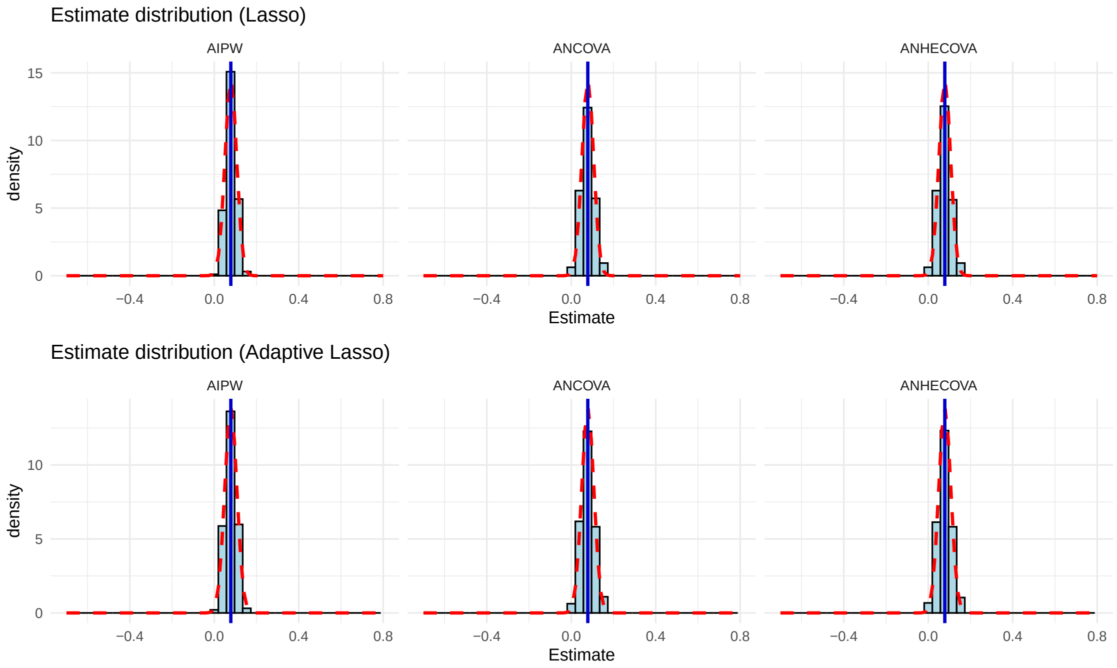

Figure B.3.16: Simulation results for estimation distributions by Lasso and adaptive Lasso variable selections, under binary outcome, nonlinear $\delta(\mathbf{X})$ and $N = 500$. The red dashed curves are the theoretical normal densities curves, and the blue lines indicate the true ATE.

References

Marlena S Bannick, Jun Shao, Jingyi Liu, Yu Du, Yanyao Yi, and Ting Ye. A general form of covariate adjustment in randomized clinical trials. *arXiv preprint arXiv:2306.10213*, 2023.

Jinyong Hahn. On the role of the propensity score in efficient semiparametric estimation of average treatment effects. *Econometrica*, pages 315–331, 1998.

Keisuke Hirano, Guido W Imbens, and Geert Ridder. Efficient estimation of average treatment effects using the estimated propensity score. *Econometrica*, 71(4):1161–1189, 2003.

Kelly Van Lancker, Iván Díaz, and Stijn Vansteelandt. Automated, efficient and model-free inference for randomized clinical trials via data-driven covariate adjustment. *arXiv preprint arXiv:2404.11150*, 2024.
